# Prognostic evaluation of quick sequential organ failure assessment score in ICU patients with sepsis across different income settings

**DOI:** 10.1186/s13054-024-04804-7

**Published:** 2024-01-23

**Authors:** Andrew Li, Lowell Ling, Hanyu Qin, Yaseen M. Arabi, Sheila Nainan Myatra, Moritoki Egi, Je Hyeong Kim, Mohd Basri Mat Nor, Do Ngoc Son, Wen-Feng Fang, Bambang Wahyuprajitno, Madiha Hashmi, Mohammad Omar Faruq, Boonsong Patjanasoontorn, Maher Jaffer Al Bahrani, Babu Raja Shrestha, Ujma Shrestha, Khalid Mahmood Khan Nafees, Kyi Kyi Sann, Jose Emmanuel M. Palo, Naranpurev Mendsaikhan, Aidos Konkayev, Khamsay Detleuxay, Yiong Huak Chan, Bin Du, Jigeeshu Vasishtha Divatia, Younsuck Koh, Jason Phua, the MOSAICS II Study Group, Uzzal Kumar Mallick, Motiul Islam, Tarequl Hamid, A. K. M. Shirazul Islam, Rabiul Halim, Md Arifur Rahman Khan, Mohammad Asaduzzaman, Md Rezaul Karim, Nahim Sarwar, Shamsul Hoque Milon, Rashed Mahmud, A. K. M. Sirajul Islam Hirok, Ashraful Haque, Amina Sultana, Mir Atiqur Rahman Shajal, Farha Andalib, Rashedul Hasan, Khalid Mahmood Khan Nafees, Shah Sudhirchandra Dhansukhlal, Ning Li, Xiaowei Liu, Haiwei Yang, Ming Hou, Ying Li, Jian Zhang, Lifeng Huang, Wenxiong Li, Meili Duan, Taotao Liu, Wei He, Fangyu Ning, Xiaozhi Wang, Xiaoyan Zhou, Sun Yu, Xiang Xiang, Liang Pan, Feihu Zhou, Yaoli Wang, Jian Zhou, Tao Wang, Xuefei Yang, Yu Ma, Xuan Song, Haiying Wu, Chuanyun Qian, Lixin Zhou, Zuohang Xu, Kun Zhang, Zhenjie Hu, Xingsheng Lin, Songjing Shi, Xiaoguang Zhang, Rongguo Yu, Liqin Zhang, Yuan Yuan, Huiru Zhou, Xiandong Wang, Zhonghua Wang, Tiehe Qin, Xianqing Shi, Rui Li, Zhenyang He, Xiangrong Zuo, Quan Cao, Tao He, Yuanda Sui, Tiejun Wu, Ying Xu, Qin Gu, Weizheng Shuai, Hanyu Qin, Bin Du, Hong Qiao, Shuangling Li, Guiying Dong, Xiujuan Zhao, Fengxue Zhu, Junshi Wang, Lei Huang, Tianchang Wang, Hao Wang, Siqing Ma, Zhengping Yang, Yuan Gao, Ruoming Tan, Yun Xie, Ruilan Wang, Jia Jia, Bin Zang, Jun Wang, Ling Lin, Yuwen Wu, Yunfu Wu, Penglin Ma, Yanfang Li, Li Yu, Rui Guo, Jiuzhi Zhang, Xianyao Wan, Feng Shen, Qindong Shi, Jun Xu, Qiang Fang, Shaohua Liu, Tongwen Sun, Mian Zeng, Weiyun Pan, Zhongmin Liu, Qingling Lin, Nan Wang, Jing Pang, Bin Xiong, Deliang Wen, Fuxin Kang, Liuhui Chang, Yun Sun, Jingxiao Zhang, Yongjie Yin, Liu Qing, Jiajun Sun, Nahui Li, Yongqiang Wang, Songtao Shou, Yanfen Chai, Lei Xu, Xiaobo Yang, Xuelian Liao, Xian Kang, Shuangping Zhao, Liquan Huang, Run Zhang, Renhua Sun, Chao Shen, Yan He, Fu Loi Chow, Michele Tang, Philip Lam, Esther Cham, Kin Bong Tang, Lowell Ling, Manimala Dharmangadan, Pauline Yeung Ng, Kin Ho Ling, Vincent Lau, Samir Sahu, Sharmila Chatterjee, Sushmita Basu, Zubair Umer Mohamed, Sudeep Sirga, Siddhartha Reddy Kasireddy, M. A. Aleem, Swarna Deepak Kuragayala, Sai Praveen Haranath, Nagarajan Ramakrishnan, Pravin Amin, Joanne Mascarenhas, Radhika Dash, Venkat Raman Kola, R. Vaidyanathan, Siddharth Agarwal, Pradip K. Bhattacharya, Deepak Jeswani, Parshotum Lal Gautam, Abdul Samad Ansari, Vivek Nangia, Mrinal Sircar, V. M. Balasubramani, S. Maneendra, Sanghamitra Mishra, Anjeev Kumar, Rajesh Chawla, Trevor Francis Sequeira, Om Prakash Shrivastava, T. V. Sreevalsan, Rajesh Mohan Shetty, Manjunath Thimmappa, M. M. Harish, Yatin Mehta, Divya Saxena, Vipul Mishra, Rishi Kumar, Simnt Kumar Jha, Prashant Sakhavalkar, Dnyaneshwar Diwane, Subhal Dixit, Kalaiselvan, Manoranjan Pattnaik, Lalit Singh, Fareed Khan, Mehul Shah, Prasanna, Ziokov Joshi, Sheila Ninan Myatra, Manoj Gorade, Bharat G. Jagiasi, Amol Hartalkar, B. Saroj Kumar Prusty, Yogesh, Ade Winata, Maulydia, Surya Oto Wijaya, Hermin Prihartini, Shinta V. R. Hutajulu, Rudy Manalu, Christrijogo Sumartono, Chrisma Adryana Albandjar, Ira Pitaloka, Dewi Kusumawati, Arifin, Akhmad Yun Jufan, Bambang Pujo Semedi, Vanessy Theodora Silalahi, Yudianto, Erwin Pradian, Achsanuddin Hanafie, Mariza Fitriati, Tinni Trihartini Maskoen, Satriawan Abadi, Calcarina Fitriani Retno Wisudarti, Johan Arifin, Reza Widyanto Sudjud, Prananda Surya Airlangga, Rupi’i, I. Made Wiryana, Anang Achmadi, Patra Rijalul Harly, Edward Kusuma, Primartanto Wibowo, Ade Veronica HY, Jeni Sarah Mandang, Meriwijanti, I. Wayan Aryabiantara, Faisal Muchtar, Fachrul Jamal Isa, Dita Aditianingsiih, Nicolaas Parningotan Simamora, Moch. Hasyim, I. Gusti Putu Manuaba, Novita Anggraeni, Rudy Ariyanto Sanoesi, Arief Munandar, Duma Saurma Siahaan, Sri Rachmawati, Oky Susianto, Liliriawati Ananta Kahar, Zulkifli, Mordekhai Leopold Laihad, Nakada Takaaki, Yoshitaka Hara, Osamu Nishida, Kenji Uehara, Makoto Takatori, Shinichiro Ohshimo, Kazuya Kikutani, Nobuaki Shime, Shin Nunomiya, Shinshu Katayama, Bengo Atari, Takashi Ito, Yasuyuki Kakihana, Kohei Takimoto, Machi Yanai, Moritoki Egi, Tomoaki Yatabe, Yuki Kishiara, Ushio Higashijima, Motohiro Sekino, Kazuaki Atagi, Hiroshi Ogura, Tsunehiro Matsubara, Tadashi Kamio, Shigeki Fujitani, Toru Yoshida, Yukari Aoyagi, Shigehiko Uchino, Masatsugu Hasegawa, Jun Oto, Naoki Yamaguchi, Yuki Enomoto, Masaki Nakane, G. S. Amirova, Murat Daribaev, Markov Viktor Evgenievich, A. A. Vorobiev, A. V. Andrushenko, Aliya Torpakbaeva, M. E. Konkayeva, A. V. Galkin, P. A. Ostanin, Khamsay Detleuxay, Noryani Mohd Samat, Ismail Tan, Nahla Irtiza Ismail, Chew Har Lim, Wan Nasrudin Wan Ismail, Siti Rohayah Sulaiman, Anita Alias, Joanne Tiong Jia Wen, Azmin Huda Abdul Rahim, Asmah Zainudin, Nik Azman Nik Adib, Zihni Abdullah, Hafizah, Mohd Zulfakar Mazlan, Mohd Basri Mat Nor, Munkhasiakhan, Naranpurev, Cho Myint Tun, Thinzar Maw, Cho Cho, Han Sein, Myo Malar Win, Lwin Lwin Hnin, Cho Cho Lwin, Aye Su Mon, Yi Sandar Thein, Khin Le Le Yi, Myo Min Naing, Nu Nu May, Lun Naing, Khin Saw Yu Aung, Moe Thu Lin, Aung Kyi, Kyaw Min Min Tun, Suu New Khin, Khin Pyone Yi, Khin May Waan, Moe Thidar, Kyi Kyi Sann, Mu Mu Naing, Win Win Mar, Naing Naing Lin, Lalit Rajbanshi, Trishant Limbu, Baburaja Shrestha, Ujma Shrestha, Ashish Shrestha, Rosi Pradhan, Ravi Ram Shrestha, Sulav Acharya, Pramesh Sunder Shrestha, Puja Thapa Karki, Moosa Awladthani, Jacob Paul, Nadia Al Badi, Adil Al Kharusi, Khalil Al Kharousi, Sandeep Kantor, Yohannan John, Said Al Mandhari, Geetha Jacob, Amr Muhammad Esmat, B. M. J. Shetty, Ahmed Mostafa, Naveed Haroon Rashid, Muhammad Sohaib, Sonia Joseph, Safia Zafar, Ahmed Farooq, Muhammad Sheharyar Ashraf, Tanveer Hussain, Muhammad Hayat, Ataur Rehman, Syed Muneeb Ali, Saad ur Rehman, Ashok Kumar, Aaron Hernandez, Crystal Aperocho, Raymundo Resurreccion, Debbie Noblezada-Uy, Jose Emmanuel Palo, Julie Visperas, Amer Asiri, Ali Beshabshi, Fahad Al-Hameed, Ohoud Al Orabi, Yaseen Arabi, Eman Al Qasim, Masood Iqbal, Tharwat Aisa, Mohammed Saeed Al Shahrani, Laila Asonto, Ayman Kharaba, Abdullah al Mutairi, Khaild Al Ghamdi, Lama Hefni, Ahmad Al Qurashi, Galeb Al Makhlafi, Roshni Sadashiv Gokhale, Noelle Lim, Manjit Pawar, Venkatesan Kumaresh, Naville Chia Chi Hock, Tan Chee Keat, Tan Rou An, Jared De Souza, Andrew Li, Yip Hwee Seng, Jason Phua, Addy Tan YH, Melvin Tay Chee Kiang, Ng Shin Yi, Ho Vui Kian, Kiran Sharma, Sennen Lew, Lee Rui Min, Do Wan Kim, Yoon Mi Shin, Song-I. Lee, Kyung Chan Kim, Yun-Seong Kang, Soo Hwan Lee, Ho Cheol Kim, Yun Su Sim, Sunghoon Park, Tai Sun Park, Hongyeul Lee, Youjin Chang, Heung Bum Lee, Je Hyeong Kim, Young Seok Lee, Won Gun Kwack, In Byung Kim, Tae Yun Park, Young Jae Cho, Sang-Min Lee, Kyeongman Jeon, Jongmin Lee, Shin Young Kim, Jin-Won Huh, Jong Joon Ahn, Jae Hwa Cho, Won-Yeon Lee, Chin-Kuo Lin, Chang-Ke Chu, Jiun-Ting Wu, Chiung-Yu Lin, Yu-Mu Chen, Kuo-Tung Huang, Han-Chung Hu, Cong-tat Cia, Jung-Yien Chien, Chun-Te Huang, Pin-Kuei Fu, Nattachai Srisawas, Manasnun Kongwibulwut, Kaweesak Chittawatanarat, Worapot Daewtrakulchai, Anakapong Phunmanee, Anupol Panitchote, Boonsong Patjanasoontorn, Chaiwut Sawawiboon, Lê Minh Trung, Đỗ Ngọc Sơn, B. S. Bùi Nhật Hà, Dương Thiện Phước, Huỳnh Quang Đại, Nguyễn Tấn Hùng, Lê Thị Phương Thúy, Hoàng Bùi Hải, Hoàng Trọng Ái Quốc, Trần Hoài Linh, Vũ Hải Yến, Phạm Trà Giang, Nguyễn Thị Ngà, Nguyễn Đăng Tuân, for the Asian Critical Care Clinical Trials Group

**Affiliations:** 1 Division of Respiratory and Critical Care Medicine, Department of Medicine, National University Hospital, National University Health System, Singapore, Singapore; 2 Department of Anaesthesia and Intensive Care, The Chinese University of Hong Kong, Hong Kong SAR, China; 3 State Key Laboratory of Complex, Severe and Rare Disease, Medical Intensive Care Unit, Peking Union Medical College Hospital, Beijing, China; 4 King Saud Bin Abdulaziz University for Health Sciences, King Abdullah International Medical Research Center, King Abdulaziz Medical City, Riyadh, Kingdom of Saudi Arabia; 5 Department of Anaesthesiology, Critical Care and Pain, Tata Memorial Hospital, Homi Bhabha National Institute, Mumbai, India; 6 Department of Anesthesiology and Intensive Care, Kyoto University Hospital, Kyoto, Japan; 7 Division of Pulmonary and Critical Care Medicine, Department of Internal Medicine, Korea University Ansan Hospital, Korea University College of Medicine, Ansan, Republic of Korea; 8 International Islamic University Malaysia Medical Center, Kuantan, Malaysia; 9 Center of Critical Care Medicine, Bach Mai Hospital, Hanoi Medical University, VNU University of Medicine and Pharmacy, Hanoi, Vietnam; 10 Division of Pulmonary and Critical Care Medicine, Department of Internal Medicine, Kaohsiung Chang Gung Memorial Hospital, Chang Gung University College of Medicine, Kaohsiung, Taiwan; 11 Department of Respiratory Care, Chang Gung University of Science and Technology, Chiayi, Taiwan; 12 Department of Anesthesiology and Reanimation, Faculty of Medicine, University of Airlangga, Intensive Care Unit, Dr Soetomo General Hospital, Surabaya, Indonesia; 13 Department of Anaesthesiology, Aga Khan University, Karachi, Pakistan; 14 General Intensive Care Unity and Emergency Department, United Hospital Ltd, Dhaka, Bangladesh; 15 Division of Respiratory and Critical Care Medicine, Department of Internal Medicine, Faculty of Medicine, Khon Kaen University, Khon Kaen, Thailand; 16 Department of Anesthesia and Critical Care, Royal Hospital, Muscat, Oman; 17 Department of Anesthesia and Intensive Care, Kathmandu Medical College Teaching Hospital, Kathmandu, Nepal; 18 RIPAS Hospital, Bandar Seri Begawan, Brunei Darussalam; 19 Department of Anaesthesiology and ICU, Yangon General Hospital, University of Medicine 1, Yangon, Myanmar; 20 Acute and Critical Care Institute, The Medical City, Pasig City, Philippines; 21 Mongolia Japan Hospital, Mongolian National University of Medical Sciences, Ulaanbaatar, Mongolia; 22 Anaesthesiology and Intensive Care Department, Astana Medical University, Astana, Kazakhstan; 23 Anaesthesiology and Intensive Care Department, National Scientific Center of Traumatology and Orthopedia Named After Academician N.D. Batpenov, Astana, Kazakhstan; 24 Adult Intensive Care Unit, Mahosot Hospital, Vientiane, Lao PDR; 25 Biostatistics Unit, Yong Loo Lin School of Medicine, National University of Singapore, Singapore, Singapore; 26 Department of Pulmonary and Critical Care Medicine, Asan Medical Center, University of Ulsan College of Medicine, Seoul, South Korea; 27 FAST and Chronic Programmed, Alexandra Hospital, National University Health System, Singapore, Singapore

**Keywords:** qSOFA, APACHE, Prediction, Critical care, Infection, Mortality

## Abstract

**Background:**

There is conflicting evidence on association between quick sequential organ failure assessment (qSOFA) and sepsis mortality in ICU patients. The primary aim of this study was to determine the association between qSOFA and 28-day mortality in ICU patients admitted for sepsis. Association of qSOFA with early (3-day), medium (28-day), late (90-day) mortality was assessed in low and lower middle income (LLMIC), upper middle income (UMIC) and high income (HIC) countries/regions.

**Methods:**

This was a secondary analysis of the MOSAICS II study, an international prospective observational study on sepsis epidemiology in Asian ICUs. Associations between qSOFA at ICU admission and mortality were separately assessed in LLMIC, UMIC and HIC countries/regions. Modified Poisson regression was used to determine the adjusted relative risk (RR) of qSOFA score on mortality at 28 days with adjustments for confounders identified in the MOSAICS II study.

**Results:**

Among the MOSAICS II study cohort of 4980 patients, 4826 patients from 343 ICUs and 22 countries were included in this secondary analysis. Higher qSOFA was associated with increasing 28-day mortality, but this was only observed in LLMIC (*p* < 0.001) and UMIC (*p* < 0.001) and not HIC (*p* = 0.220) countries/regions. Similarly, higher 90-day mortality was associated with increased qSOFA in LLMIC (*p* < 0.001) and UMIC (*p* < 0.001) only. In contrast, higher 3-day mortality with increasing qSOFA score was observed across all income countries/regions (*p* < 0.001). Multivariate analysis showed that qSOFA remained associated with 28-day mortality (adjusted RR 1.09 (1.00–1.18), *p* = 0.038) even after adjustments for covariates including APACHE II, SOFA, income country/region and administration of antibiotics within 3 h.

**Conclusions:**

qSOFA was independently associated with 28-day mortality in ICU patients admitted for sepsis. In LLMIC and UMIC countries/regions, qSOFA was associated with early to late mortality but only early mortality in HIC countries/regions.

**Graphical Abstract:**

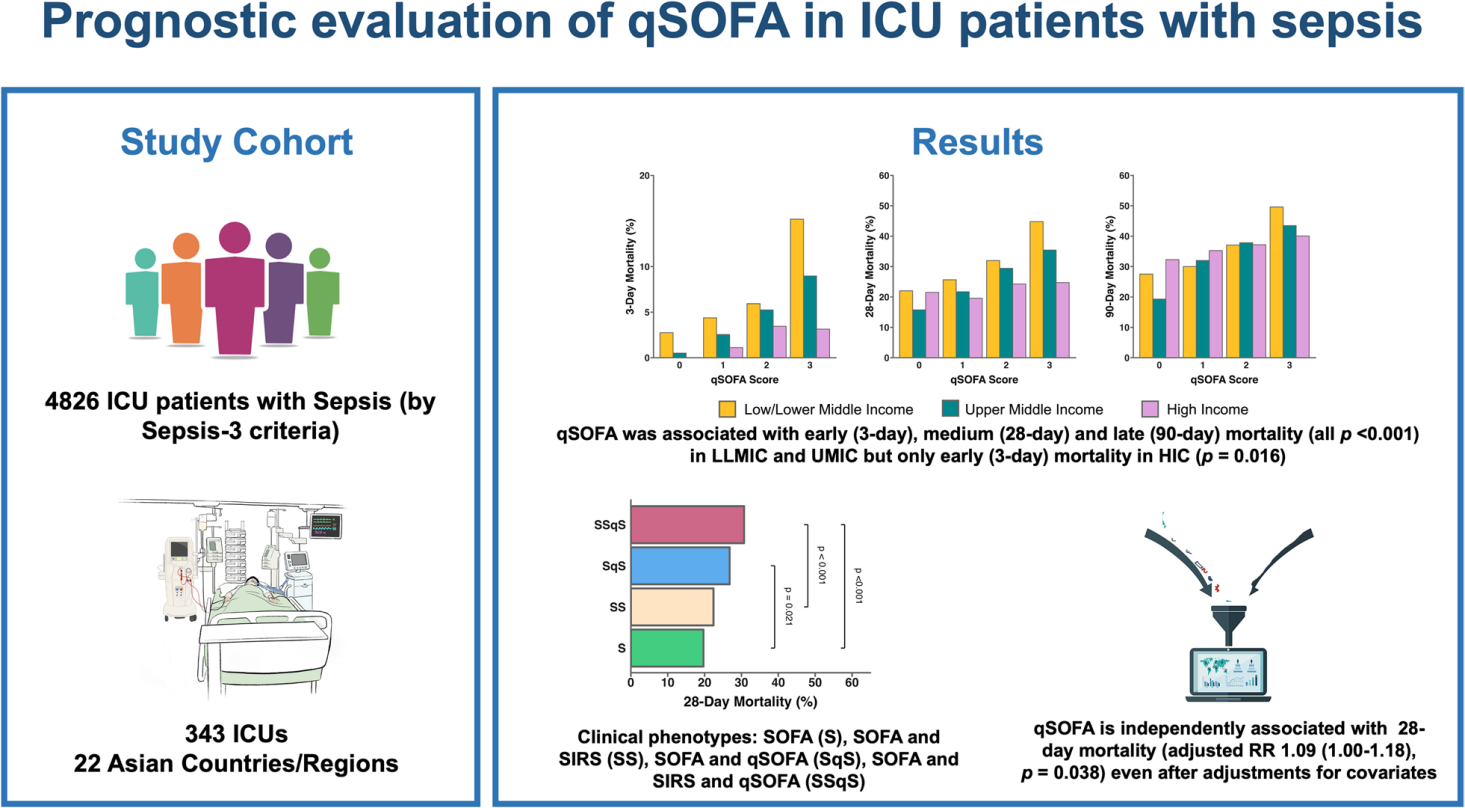

**Supplementary Information:**

The online version contains supplementary material available at 10.1186/s13054-024-04804-7.

## Introduction

Sepsis-3 consensus definitions introduced quick Sequential Organ Failure Assessment (qSOFA) as a sepsis screening tool to identify patients with higher hospital mortality [1]. The original Sepsis-3 derivation cohort showed that Sequential Organ Failure Assessment (SOFA) was superior when compared to qSOFA or systemic inflammatory response syndrome (SIRS) for prediction of mortality in the intensive care unit (ICU) [2]. Subsequent early validation studies on qSOFA from ICUs in high income settings have confirmed these limitations [3–6]. Thus, its potential role as a simple sepsis diagnostic and outcome prediction tool has been mostly evaluated in the Emergency Department [7–9].

Whether qSOFA has prognostic value for patients with sepsis in low resourced ICUs where Acute Physiology and Chronic Health Evaluation (APACHE) II score or SOFA may not be practical has not been robustly assessed [10, 11]. Recent evidence from low resource settings does suggest qSOFA is associated with mortality in hospitalized patients with infection [12–16].The addition of lactate has also been shown to improve qSOFA sepsis mortality prediction in all income settings [17–21].

Studies on qSOFA mortality prediction in ICU patients with sepsis have generally used all-cause hospital mortality as the endpoint. However, since calculation of qSOFA is based on physiological parameters alone, its predictive performance is likely more dependent on time from assessment than severity scores such as APACHE II that incorporate variables on chronic health. This has been demonstrated in a large retrospective study in Taiwan which showed that the association between qSOFA mortality was greater at 72 h than 28-days [22].

The Management of Severe sepsis in Asia’s Intensive Care unitS (MOSAICS) II was a prospective, observational, cross-sectional point prevalence study on sepsis epidemiology in 386 ICUs from 22 Asian countries/regions across all income settings [23]. Detailed admission physiological data was collected from a well-defined cohort of 4980 ICU patients admitted for sepsis. In this secondary analysis of MOSAICS II, our primary aim was to determine the association between qSOFA and 28-day mortality in ICU patients admitted for sepsis. Association of qSOFA with early (3-day), medium (28-day), late (90-day) mortality was assessed in low and lower middle income (LLMIC), upper middle income (UMIC) and high income (HIC) countries/regions. Predictive performance of qSOFA on 28-day mortality was compared to APACHE II, SIRS, SOFA. Additional value of lactate measurement on qSOFA sepsis mortality prediction was assessed. Lastly, we described the characteristics and outcomes of different clinical sepsis phenotypes in patients who met Sepsis-3 SOFA criteria and simultaneously fulfilled SIRS and qSOFA criteria.

## Methods

### Study design and score criteria

The MOSAICS II study recruited adult patients (age ≥ 18 years old) who were admitted to the ICU for treatment of sepsis. Sepsis was defined using Sepsis-3 criteria as infection with a ∆SOFA ≥ 2 from baseline [1]. They were recruited on four separate days of different quarters in 2019 [23]. All patients from the MOSAICS II cohort who had ICU admission qSOFA recorded were included in this study. Patients who had missing SOFA, APACHE II and survival status at 90 days were excluded from this analysis. Countries and regions were grouped by income according to the 2019 World Bank Classification [24].

### Score criteria

Physiological data and laboratory results closest to time of ICU admission was used to calculate ∆SOFA, SIRS and qSOFA score. Change in SOFA at ICU admission from baseline SOFA (assumed to be zero if without prior organ dysfunction) was calculated to obtain ∆SOFA score (0 to 24 points) [25]. qSOFA score (0 to 3 points) was calculated by summation of individual components with 1 point each for SBP < 100 mmHg, respiratory rate ≥ 22 breaths/min and altered mental status (Glasgow Coma Scale < 15) [1, 3, 4]. SIRS (0 to 4 points) was assessed by fulfillment of four individual components: heart rate > 90 beats/min; temperature < 36 °C or > 38 °C; white blood cell count > 12,000/μL or < 4000/μL; respiratory rate > 20 breaths/min or arterial PCO_2_ < 32 mmHg [26]. Thresholds to fulfill score criteria were ∆SOFA ≥ 2 for SOFA, ≥ 2 for SIRS, ≥ 2 for qSOFA [1, 26].

### Mortality endpoint

All patients recruited to MOSAICS II were prospectively followed up for all-cause mortality from initial time of sepsis recognition until day 90. Patients who were alive but not discharged from hospital at 90 days were classified as survivors at 90 days. Early (3-day), medium (28-day) and late (90-day) mortality was assessed.

### Predictive performance of scores

Predictive performances of APACHE II, SOFA, SIRS, qSOFA and qSOFA with lactate to predict early to late mortality were assessed using area under the receiver operating characteristic curve (AUC), sensitivity and specificity. The lactate value obtained closet to time of ICU admission (within 24 h) was used for analysis. Patients who did not have lactate results were excluded in this part of the analysis. The thresholds selected for each score was determined for each mortality timepoint using Youden’s index. A subgroup analysis excluding patients who remained in hospital by day 90 was performed to evaluate the discriminatory performance of each score for hospital mortality.

### Clinical sepsis phenotype

Patients were classified into four clinical sepsis phenotype groups accordingly: “SOFA only” (S), “SOFA and SIRS” (SS), “SOFA and qSOFA” (SqS) and “SOFA and SIRS and qSOFA” (SSqS). Phenotype S consisted of patients who only had SOFA without fulfilling SIRS or qSOFA criteria. Phenotype SS consisted of those who fulfilled both SOFA and SIRS criteria but had qSOFA < 2. Phenotype SqS consisted of patients with sepsis who fulfilled both SOFA and qSOFA criteria but had SIRS < 2. Those who met the thresholds for all three scores were included in the SSqS phenotype.

### Statistical analysis

Shapiro–Wilk test was used to test for normality of numerical variables. Descriptive statistics using proportions and median with interquartile range were used to summarize data. Kruskal–Wallis and ϰ^2^ tests were used to assess the differences in characteristics between clinical phenotype groups. Pairwise ϰ^2^ test was used to assess differences in mortality between different clinical phenotypes. Association between overall qSOFA score and individual qSOFA components were compared within each income region/country using ϰ^2^ test. Fischer’s test was used instead of ϰ^2^ test when appropriate.

Multivariate analysis using modified Poisson regression was used to determine the adjusted relative risk (RR) of qSOFA score on mortality at 28 days. A directed acyclic graph was used to select covariates from identified confounders on hospital mortality in MOSAICS II and other illness severity scores [23]. These included income region/country, age, sex, solid malignant tumor, immunosuppression, hematological malignancy, Emergency Department admission, unscheduled surgical admission, antibiotic within 3 h of sepsis recognition, APACHE II and SOFA (see Additional file 1: Fig. S1). Additional multivariate analyzes were performed to assess the association between qSOFA and mortality at 3-days and 90-days.

Difference in AUC on 28-day mortality prediction between scores were compared using Delong’s method. McNemar’s test was used to evaluate differences in sensitivities and specificities of severity scores. Significance value was set at *α* < 0.05. Data analyzes were performed in R studio (v 2023.06.1 + 524) with reportROC and ggplot packages.

## Results

### Cohort characteristics

Among the MOSAICS II study cohort of 4980 patients, 4826 patients from 343 ICUs and 22 countries were included in this secondary analysis (see Additional file 1: Fig. S2). The baseline characteristics of included patients grouped by clinical phenotype are shown in Table 1. Overall, 696/4826 (14.4%) fulfilled only SOFA criteria, 1495/4826 (31.0%) fulfilled both SOFA and SIRS, 265/4826 (5.5%) fulfilled both SOFA and qSOFA, and 2370/4826 (49.1%) fulfilled all of SOFA, SIRS and qSOFA criteria. The proportion of individual qSOFA score components in different income countries/regions are shown in Fig. S3, Additional file 1. There were 281 patients who remained in hospital at 90 days. HIC countries/regions had the highest proportion of patients who remained in hospital by day 90 compared to LLMIC and UMIC countries/regions (*p* < 0.001) (see Additional file 1: Fig. S4 and Fig. S5).

**Table 1:** Baseline Characteristics by Clinical Sepsis Phenotype

	S (*n* = 696)	SS (*n* = 1495)	SqS (*n* = 265)	SSqS (*n* = 2370)	Overall (*n* = 4826)	*p*
Age	68 [55, 79]	62 [49,72]	68 [57, 78]	65 [51, 76]	64.0 [51, 76]	0.211
Male Sex (%)	427 (61.4)	904 (60.5)	159 (60.0)	1486 (62.7)	2976 (61.7)	0.509
Income Country/Region (%)						
Low/Lower Middle Income	167 (24.0)	445 (29.8)	77 (29.1)	780 (32.9)	1469 (30.4)	< 0.001
Upper Middle Income	317 (45.5)	627 (41.9)	94 (35.5)	816 (34.4)	1854 (38.4)	
High Income	212 (30.5)	423 (28.3)	94 (35.5)	774 (32.7)	1503 (31.1)	
Positive Microbiology (%)	474 (68.1)	955 (63.9)	183 (69.1)	1670 (70.5)	3282 (68.0)	< 0.001
Antibiotics within 3 Hours (%)	481 (69.1)	1110 (74.2)	198 (74.7)	1691 (71.4)	3480 (72.1)	0.139
SIRS	1 [0, 1]	2 [2, 3]	1 [1]	3 [2, 3]	2 [2, 3]	< 0.001
qSOFA	1 [0, 1]	1 [1]	2 [2]	2 [2, 3]	2 [1, 2]	< 0.001
SOFA	6 [4, 9]	6 [3, 9]	8 [5, 11]	8 [6, 11]	7 [4, 10]	< 0.001
APACHE II	17 [12, 22]	17 [12, 22]	21 [15, 28]	22 [17, 28]	20 [14, 26]	< 0.001
Lactate	1.6 [1.1, 2.8]	2.1 [1.3, 3.6]	2.1 [1.2, 3.5]	2.8 [1.6, 5]	2.3 [1.4, 4.2]	< 0.001
Vasopressors (%)	420 (60.3)	907 (60.7)	200 (75.5)	1818 (76.7)	3345 (69.3)	< 0.001
MV (%)	507 (72.8)	1008 (67.4)	204 (77.0)	1824 (77.0)	3543 (73.4)	< 0.001
RRT (%)	198 (28.4)	443 (29.6)	85 (32.1)	790 (33.3)	1516 (31.4)	0.025
ICU LOS, days	13 [6, 27]	11 [5, 22]	13 [6, 29]	11 [6, 23]	12 [6, 23]	0.426
Hospital LOS, days	23 [13, 43]	21 [11, 37]	24 [13, 41]	20 [11, 38]	21 [11, 38]	0.179

Phenotype S consisted of patients who only had SOFA without fulfilling SIRS or qSOFA criteria. Phenotype SS consisted of those who fulfilled both SOFA and SIRS criteria but had qSOFA < 2. Phenotype SqS consisted of patients with sepsis who fulfilled both SOFA and qSOFA criteria but had SIRS < 2. Those who met the thresholds for all three scores were included in the SSqS phenotype. Values are expressed as median and interquartile range unless otherwise specified

APACHE, Acute Physiology And Chronic Health Evaluation; ICU, intensive care unit; LOS, length of stay; MV, mechanical ventilation; qSOFA, quick sequential organ failure assessment; SOFA, sequential organ failure assessment; RRT, renal replacement therapy

### qSOFA and associated mortality

**Fig. 1:**
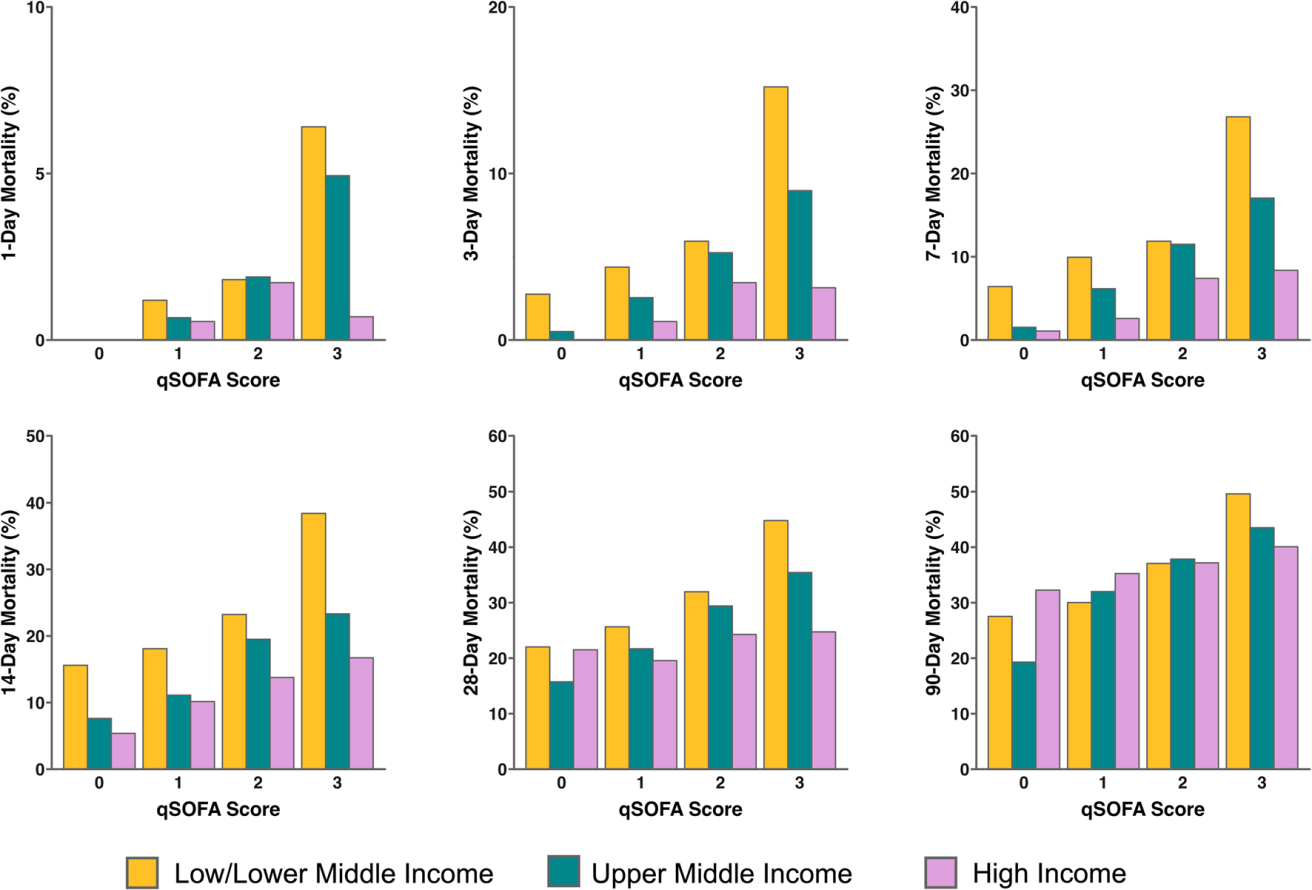
Mortality associated with qSOFA score. Legend: All-cause mortality risk calculated from time of sepsis diagnosis to time of death. qSOFA, quick sequential organ failure assessment

Early to late mortality rates associated with range of qSOFA scores in different income countries/regions are shown in Fig. 1. Overall, 28-day mortality progressively increased with higher qSOFA (*p* < 0.001). However, this 28-day trend was only observed in LLMIC (*p* < 0.001) and UMIC (*p* < 0.001) and not HIC (*p* = 0.220) countries/regions (see Additional file 2: Table S1). Similarly, higher 90-day mortality was associated with increased qSOFA in LLMIC (*p* < 0.001) and UMIC (*p* < 0.001) only. In contrast, higher 3-day mortality with increasing qSOFA score was observed across all income countries/regions (*p* < 0.001). Only systolic blood pressure and altered mental status components of qSOFA were consistently associated with 28-day mortality in both LLMIC and UMIC (see Additional file 2: Table S2). In contrast, none of the qSOFA components were associated with 28-day mortality in HIC countries/regions.

Multivariate analysis showed that qSOFA remained associated with 28-day mortality (adjusted RR 1.09 [1.00–1.18], *p* = 0.038) even after adjustments for covariates including APACHE II, SOFA, income country/region and administration of antibiotics within 3 h (Table 2). In addition, qSOFA was associated with 3-day mortality (adjusted RR 1.52 [1.24–1.87], *p* < 0.001) but was not associated with 90-day mortality (adjusted RR 1.03 [0.96–1.100], *p* = 0.454) on multivariate analysis (see Additional file 2: Tables S3 and S4). In contrast, APACHE II and SOFA were both independently associated with mortality at 3-days, 28-days and 90-days.

**Table 2:** Multivariate analysis of association between qSOFA and 28-Day Mortality

	Adjusted Relative Risk	*p*
Income Country/Region		
High Income	Reference	< 0.001
Upper Middle Income	1.17 (1.00–1.37)	
Low/Lower Middle Income	1.76 (1.50–2.07)	
Age	1.00 (1.00–1.01)	0.332
Female Sex	0.95 (0.84–1.08)	0.438
Solid Malignant Tumor	1.48 (1.24–1.74)	< 0.001
Immunosuppression	1.16 (0.88–1.5)	0.276
Hematological Malignancy	1.51 (1.15–1.96)	0.004
Emergency Department Admission	1.01 (0.89–1.15)	0.863
Unscheduled Surgical Admission	0.94 (0.77–1.14)	0.533
Antibiotics within 3 Hours	0.61 (0.53–0.70)	< 0.001
APACHE II	1.03 (1.02–1.03)	< 0.001
qSOFA	1.09 (1.00–1.18)	0.038
SOFA	1.03 (1.01–1.05)	< 0.001

APACHE, Acute Physiology And Chronic Health Evaluation; qSOFA, quick sequential organ failure assessment; SOFA, sequential organ failure assessment

### Mortality prediction performance of APACHEII, SOFA, SIRS, qSOFA

**Fig. 2:**
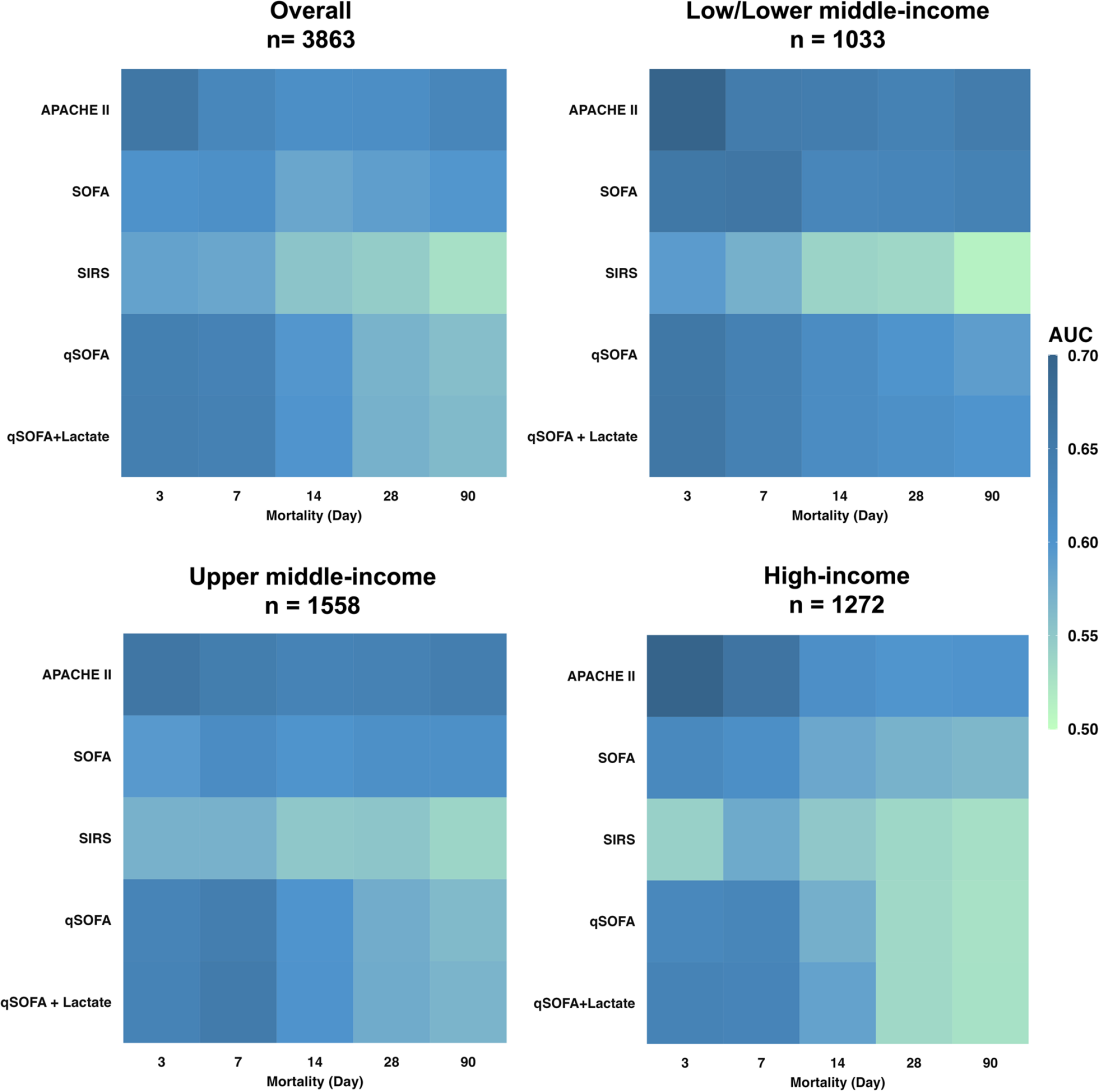
Mortality predictive performance of different scores across income regions/countries. Heatmap showing the area under the curve (AUC) of APACHE II, SOFA, SIRS, qSOFA and qSOFA with lactate to predict mortality across all income countries/regions. APACHE, Acute Physiology And Chronic Health Evaluation; qSOFA, quick sequential organ failure assessment; SIRS, systemic inflammatory response syndrome; SOFA, sequential organ failure assessment

Predictive performance of APACHE II, SOFA, SIRS, qSOFA, and qSOFA with lactate for mortality were evaluated in 3,863 patients after exclusion of 1091 patients who did not have lactate results within 24 h of ICU admission. The AUCs of different scores on prediction of short to long term mortality are shown in Fig. 2. The addition of lactate did not improve the AUC of qSOFA to predict 28-day mortality (qSOFA with lactate [0.644 (95%CI 0.602–0.685)] vs. qSOFA [0.642 (95%CI 0.601–0.682)], *p* = 0.175) (see Additional file 2: Table S5). On subgroup analysis, there was no difference between qSOFA with lactate and qSOFA alone on prediction of 28-day mortality in any of the income countries/regions (see Additional file 1: Fig. S6 and Additional file 2: Tables S6–S8).

qSOFA had lower AUC compared to APACHE II to predict 28-day mortality in HIC region/countries (APACHE II [0.599 (95%CI 0.562–0.636)] vs. qSOFA [0.530 (95%CI 0.495–0.566)], *p* = 0.001) and UMIC region/countries (APACHE II [0.636 (95%CI 0.604–0.669)] vs. qSOFA [0.576 (95%CI 0.545–0.608)], *p* < 0.001). In contrast, qSOFA had similar AUC compared to APACHE II predicted 28-day mortality in LLMIC (APACHE II 0.637 (95%CI 0.601–0.674) vs. qSOFA 0.601 (95%CI 0.566–0.636), *p* = 0.077). Since qSOFA was not associated with 90-day mortality in multivariate analysis, additional testing was performed to confirm qSOFA was indeed inferior to APACHE II in 90-day mortality discrimination in LLMIC (AUC 0.591 [95%CI 0.557–0.625] vs. 0.649 [95%CI 0.614–0.683], *p* = 0.003). In contrast, there was no difference between the AUCs of qSOFA and SOFA to predict 28-day mortality in any of the income countries/regions.

Overall, APACHE II and qSOFA both had poor specificity for 28-day mortality, although APACHE II was slightly higher [0.566 (95%CI 0.547–0.584) vs 0.490 (95%CI 0.471–0.508), *p* < 0.001]. Similarly, the sensitivity of either APACHE II or qSOFA for 28-day mortality was poor for 28-day mortality [0.608 (95%CI 0.577–0.638) vs. 0.625 (95%CI 0.595–0.655), *p* = 0.363]. Comparatively, qSOFA had higher sensitivity (*p* = 0.048) but lower specificity (*p* < 0.001) compared to SOFA which had sensitivity of 0.586 (95%CI 0.555–0.617) and specificity of 0.549 (95%CI 0.531–0.567) to predict 28-day mortality. Predictive performance of different scores for 3-day, 28-day and 90-day mortality are shown in Tables S5–S8, Additional file 2. The predictive performances of different scores for hospital mortality in subgroup analysis excluding patients who remained in hospital by day 90 were similar to the performances of the scores for 90-day mortality in the whole study cohort (see Additional file 2: Tables S9–S12).

### Clinical sepsis phenotype and associated mortality

**Fig. 3:**
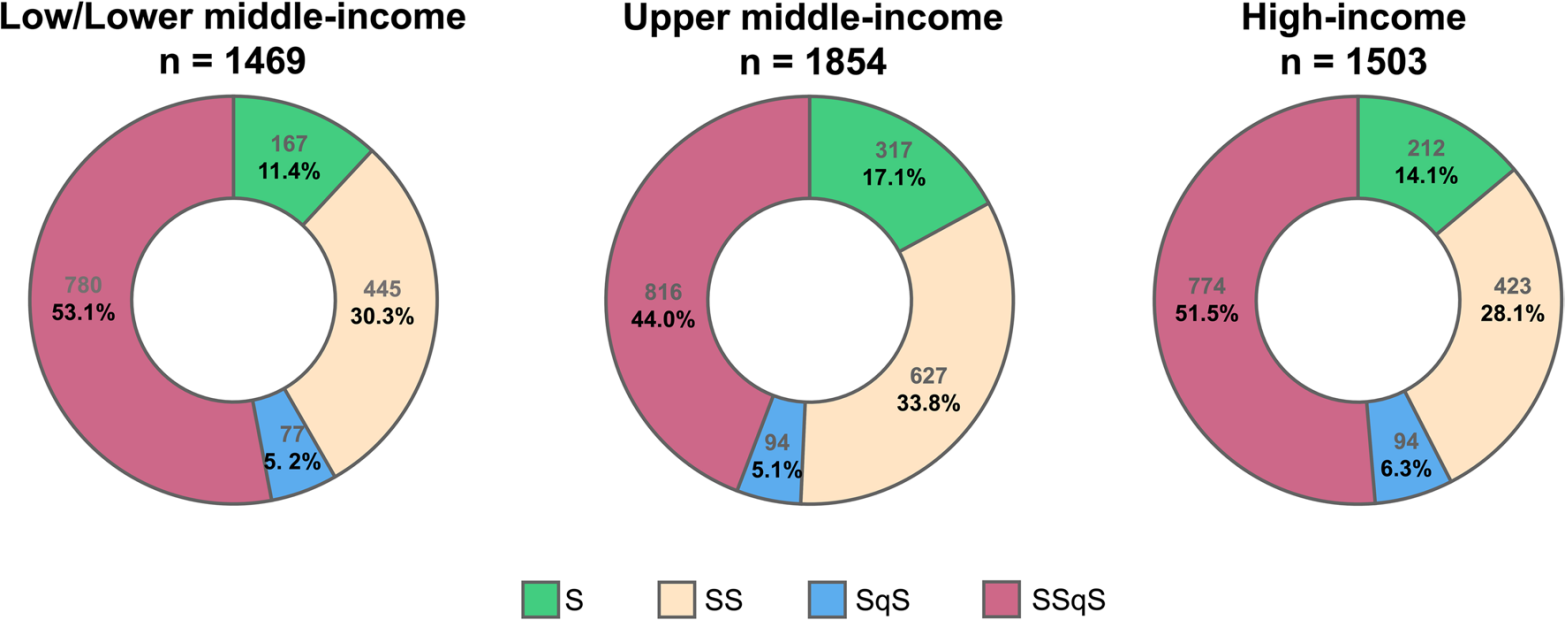
Proportion of clinical sepsis phenotype in different income countries/regions. Patients with sepsis were classified into four clinical sepsis phenotype groups according to fulflilment of score criteria: “SOFA only” (S), “SOFA and SIRS” (SS), “SOFA and qSOFA” (SqS) and “SOFA and SIRS and qSOFA” (SSqS). Distribution of patients with different clinical phenotypes across all income country/region groups. APACHE, Acute Physiology And Chronic Health Evaluation; qSOFA, quick sequential organ failure assessment; SIRS, systemic inflammatory response syndrome; SOFA, sequential organ failure assessment

**Fig. 4:**
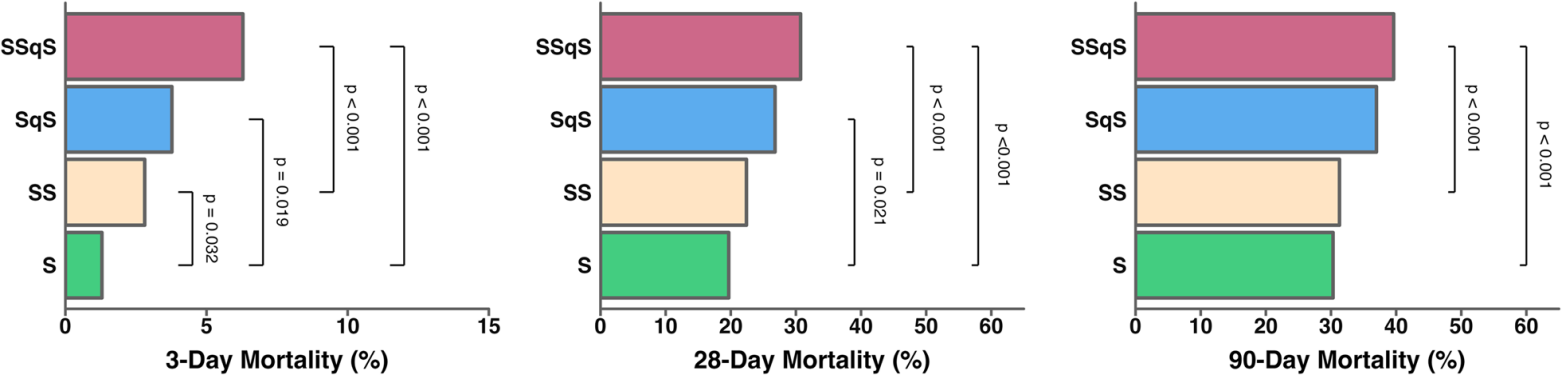
Clinical sepsis phenotype and associated all-cause mortality. Patients with sepsis were classified into four clinical sepsis phenotype groups according to fulflilment of score criteria: “SOFA only” (S), “SOFA and SIRS” (SS), “SOFA and qSOFA” (SqS) and “SOFA and SIRS and qSOFA” (SSqS). APACHE, Acute Physiology And Chronic Health Evaluation; qSOFA, quick sequential organ failure assessment; SIRS, systemic inflammatory response syndrome; SOFA, sequential organ failure assessment

SOFA and APACHE II scores were higher in phenotypes which fulfilled the qSOFA criteria (SqS and SSqS) compared to those that did not (S and SS) (Table 1). Use of vasopressors, mechanical ventilation and kidney replacement therapy were also higher phenotypes SqS and SSqS than S or SS. The proportions of each clinical sepsis phenotype in different income countries/regions are shown in Fig. 3. Overall, phenotype S had lower 28-day mortality (19.7%) when compared to those with phenotype SqS (26.8%, *p* < 0.001) or SSqS (30.7%, *p* < 0.001) (Fig. 4). Patients with the SSqS phenotype had higher 3-day and 90-day mortality when compared to both phenotype S or SS (*p* < 0.001).

## Discussion

In this secondary analysis of the MOSAICS II study, we found that qSOFA was independently associated with 28-day mortality in ICU patients admitted for sepsis. qSOFA was associated with early to late mortality in LLMIC and UMIC but only early mortality in HIC countries/regions. The 28-day mortality prediction performance of APACHE II was superior to qSOFA in HIC and UMIC. qSOFA and APACHE II had comparable, but low discriminatory performance for 28-day sepsis mortality in LLMIC. The addition of lactate did not improve the prognostic performance of qSOFA to predict 28-day sepsis mortality. The combination of SOFA, SIRS and qSOFA at time of ICU admission identified a clinical phenotype of sepsis associated with higher severity of illness and early to late mortality.

qSOFA was originally proposed as a sepsis screening tool rather than prognostic score among patients with suspected infection [1]. Moreover, interest in qSOFA as a sepsis outcome predictor in ICU patients was limited as early studies from HIC countries/regions suggest it was not better than, or even inferior to mortality prediction of SOFA [3–5]. In contrast, recent data from resource limited settings showed that qSOFA may have prognostic value even for patients with sepsis in the ICU [6, 27–29]. Our study adds clarity to these seemingly contradictory findings. Earlier study cohorts were either large retrospective cohorts or single center prospective studies from a specific income setting. Moreover, studies from limited resource settings used early mortality endpoints such as ICU mortality or 28-day mortality, whereas those from well-resourced settings assessed hospital mortality. In this study we used a well-defined, large prospective cohort of ICU patients from all income settings who met Sepsis-3 criteria to assess association between qSOFA and early (3-day) to late (90-day) sepsis mortality. We found that qSOFA was indeed associated with early to late mortality in LLMIC and UMIC but only early mortality in HIC countries/regions for ICU patients with sepsis. Furthermore, we showed that qSOFA was independently associated with 3-day and 28-day but not 90-day mortality in sepsis after adjustment for income setting. Combined with earlier studies, we conclude that association between qSOFA and sepsis mortality is both time dependent and variable across income settings.

Surprisingly, qSOFA was independently associated with 3-day and 28-day mortality even after adjustment for APACHE II and SOFA. Of note, it is interesting that qSOFA had higher adjusted RR compared to APACHE II for 3-day and 28-day mortality. Since qSOFA scoring is based on physiological variables alone, it seems reasonable that association between qSOFA and sepsis mortality was only significant in the early to medium term. Whereas for longer term survival at 90 days, age and comorbidities (adjusted for in APACHE II calculation) likely play larger roles compared to physiology predictors alone [30].

It should be noted that APACHE II, SOFA, qSOFA and SIRS all had limited discriminatory performance, sensitivity and specificity to predict sepsis mortality in ICU patients across all income settings in this study. The predictive performance of SOFA (AUC 0.569–0.630) for 28-day mortality in ICU patients with sepsis was lower in this study compared to previous reports from Thailand (AUC 0.839) or Australia and New Zealand (AUC 0.753) [3, 31]. Similarly, the mortality discrimination of APACHE II in this study (AUC 0.599–0.637) was lower than those reported from Saudi Arabia (AUC 0.782) or United States (AUC 0.80) [32, 33]. There are several reasons which may explain the observed differences. First, we calculated SOFA based on parameters closest to time of ICU admission. Instead, most studies used the worst clinical parameters within the first 24 h to calculate admission SOFA score. However, this does not explain why APACHE II also had comparatively lower predictive performance for mortality in this study. Second, our study evaluated predictive performance for 28-day mortality whereas comparative studies assessed hospital mortality. Yet, even when patients who remained in hospital at day 90 were excluded in subgroup analysis to avoid bias from censorship, discriminatory performance for hospital mortality of APACHE II and SOFA were still lower when compared to previous studies (Supplementary Table 9–12, Additoinal File 2). Third, the MOSAICS II cohort was constructed from ICU patients admitted for sepsis who all met SOFA criteria of Sepsis-3, whereas other cohorts used diagnostic coding or SIRS criteria [3, 31]. Since the discordance between SOFA, qSOFA and SIRS on diagnosis and outcomes of sepsis has been well documented, differences in study inclusion criteria for sepsis alone may have altered cohort characteristics and outcomes [14, 31, 34–37].

Although qSOFA’s predictive performance for mortality in ICU patients with sepsis was generally limited, its practical utility may differ according to income setting. Similar to data from Austrailia, we found that qSOFA was inferior to APACHE II in HIC and UMIC countries/regions on prediction of 28-day mortality [19]. In these settings, qSOFA will unlikely be used for prognostication in the ICU. However, qSOFA was comparable to APACHE II in LLMIC. Although APACHE II is widely used as a severity score for benchmarking, full calculation of APACHE II is not universally available in low resource settings [10, 11]. However, justifying use of qSOFA in low resource ICUs to provide early sepsis prognostication based on its comparable mortality discrimination to APACHE II has its own caveats. First, APACHE II was developed in HIC with limited calibration for LLMIC, and alternate scoring systems with better performance tailored for low resource settings have been proposed [11, 38]. In fact, APACHE II itself had relatively lower 28-day mortality discrimination performance (AUC 0.612) in the LLMIC cohort compared to UMIC or HIC. Severity scores may perform differently in LLMICs because of difference in case-mix with younger and minimal comorbidities when compared to HIC settings [38, 39]. These differences in patient characteristics were highlighted in the original MOSAICS II study [23]. Furthermore, MOSAICS II also showed sepsis mortality was higher in ICUs from LLMIC and UMIC when compared to HIC even after adjustment for confounders. Second, APACHE II was originally validated for predicting hospital mortality rather than specific day of mortality [40]. Alternatively, SIRS is relatively easy to compute by the bedside even in low resourced ICUs. But our results are consistent with other studies that showed SIRS underperforms when compared to qSOFA and other severity scores for mortality prediction [2, 3, 31, 41, 42].

In contrast to previous studies, addition of lactate measurement did not improve qSOFA sepsis mortality prediction [17–21]. Our discrepant results do not discount the utility of lactate measurement as there are several methodological and cohort differences between this study and previous reports. First, MOSAICS II only included ICU patients, whereas other studies were mostly focused on patients outside the ICU [18, 20, 21]. In addition, the mortality rate was much higher in this study compared to other studies. Second, the median lactate score was higher in this study. This suggests lactate may be better at discriminating patients who have very low risk of death against those with any elevated risks of death than discriminating those with moderate from very high risk of death from infection. In practice, lactate measurement is likely most useful outside the ICU setting to identify patients with sepsis at risk of higher mortality.

Value of combining SOFA, qSOFA and SIRS criteria to identify clinical phenotypes of sepsis has not been robustly evaluated. A retrospective study from China showed that hospitalized patients with sepsis who met both SOFA and qSOFA had higher hospital mortality when compared to those who only fulfilled SOFA alone [14]. We found that fulfillment of qSOFA criteria in addition to SOFA or in combination with SOFA and SIRS identified a clinical sepsis phenotype associated with higher requirements for mechanical ventilation, vasopressors and kidney replacement therapy. The SSqS phenotype which fulfilled all three criteria was associated with higher early to late mortality across all income countries/regions when compared to S phenotype (SOFA only).

The major strength our study was inclusion of prospectively collected data on patients with sepsis from 343 ICUs representing all income regions with minimal missing data and lost to follow up at 90 days. This facilitated granular comparisons on the association of qSOFA with sepsis mortality from different resource settings. This builds on the limited data supporting use of qSOFA for ICU patients in low resource settings which have been studies that were single center, had small sample sizes or included patients who only had suspected sepsis [12, 13, 29]. However, our study also has several limitations. First, because recruitment into MOSAICS II was by SOFA criteria, this study did not include patients who may have sepsis but did not meet Sepsis-3 criteria. Second, MOSAICS II was an epidemiological study and treatment was not protocolized. Third, we assessed all-cause mortality and reasons for early mortality were not documented.

## Conclusion

qSOFA was independently associated with 28-day mortality in ICU patients admitted for sepsis. In LLMIC and UMIC countries/regions, qSOFA was associated with early to late mortality but only early mortality in HIC countries/regions. Combination of SOFA, SIRS and qSOFA identified a clinical phenotype of sepsis which is associated with early to late mortality.

## Supplementary Information

**Additional file 1.** Supplementary Figures 1–6.

**Additional file 2.** Supplementary Tables 1–12.

**Additional file 3.** Appendix 1: Contributing centers listed alphabetically per country/region with Lead Investigators and Co-Investigators.

## Data Availability

The data that support the findings of this study are available from the corresponding author upon reasonable request.
