# Supplementary material for: Prognostic evaluation of quick sequential organ failure assessment score in ICU patients with sepsis across different income settings
Source: Crit Care. 2024 Jan 23;28:30. doi: 10.1186/s13054-024-04804-7 (PMC10804657; doi:10.1186/s13054-024-04804-7)
Supplement: Supplementary file 1 — Additional file 1. Supplementary Figures 1–6. [file 13054_2024_4804_MOESM1_ESM.docx]

**Supplementary Figures**

**Supplementary Figure 1: Directed acyclic graph**

**
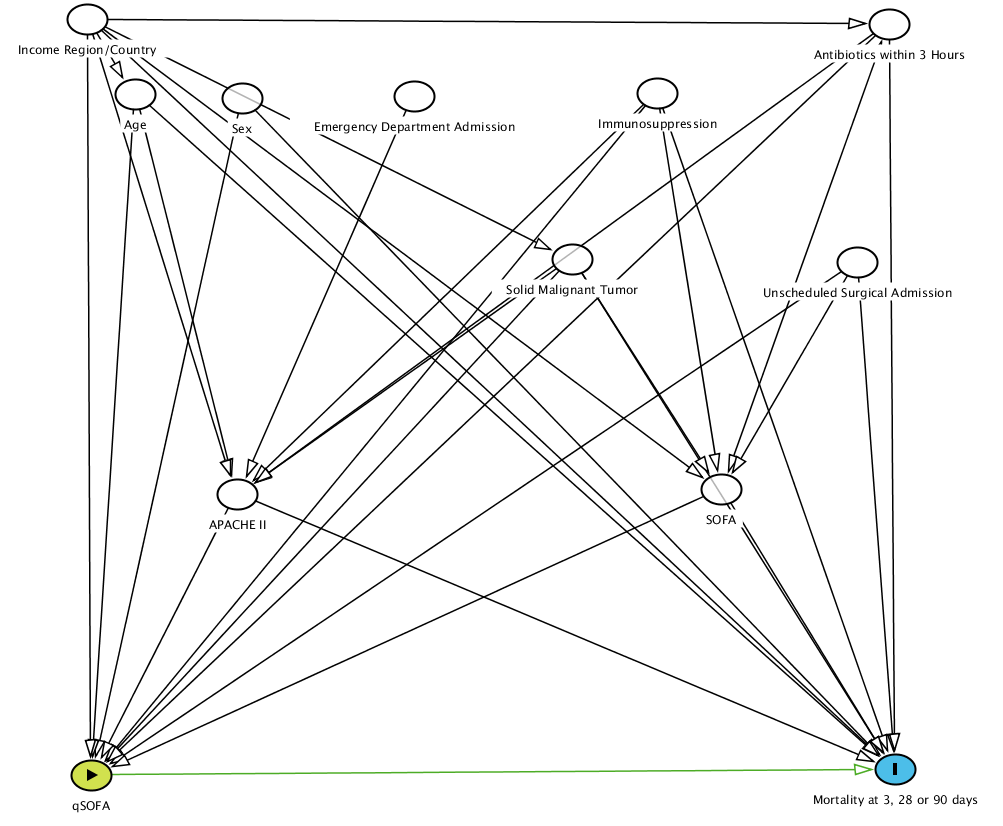
**

Minimal sufficient adjustment sets containing APACHE II, Age, Antibiotics within 3 Hours, Emergency Department Admission, Immunosuppression, Income Region/Country, SOFA, Sex, Solid Malignant Tumor, Unscheduled Surgical Admission for estimating the total effect of qSOFA (green) on Mortality (blue) at 3, 28 or 90 days: APACHE II, Age, Antibiotics within 3 Hours, Emergency Department Admission, Immunosuppression, Income Region/Country, SOFA, Sex, Solid Malignant Tumor, Unscheduled Surgical Admission. APACHE, Acute Physiology And Chronic Health Evaluation; qSOFA, quick sequential organ failure assessment; SOFA, sequential organ failure assessment.

**Supplementary Figure 2: Study Flow Chart
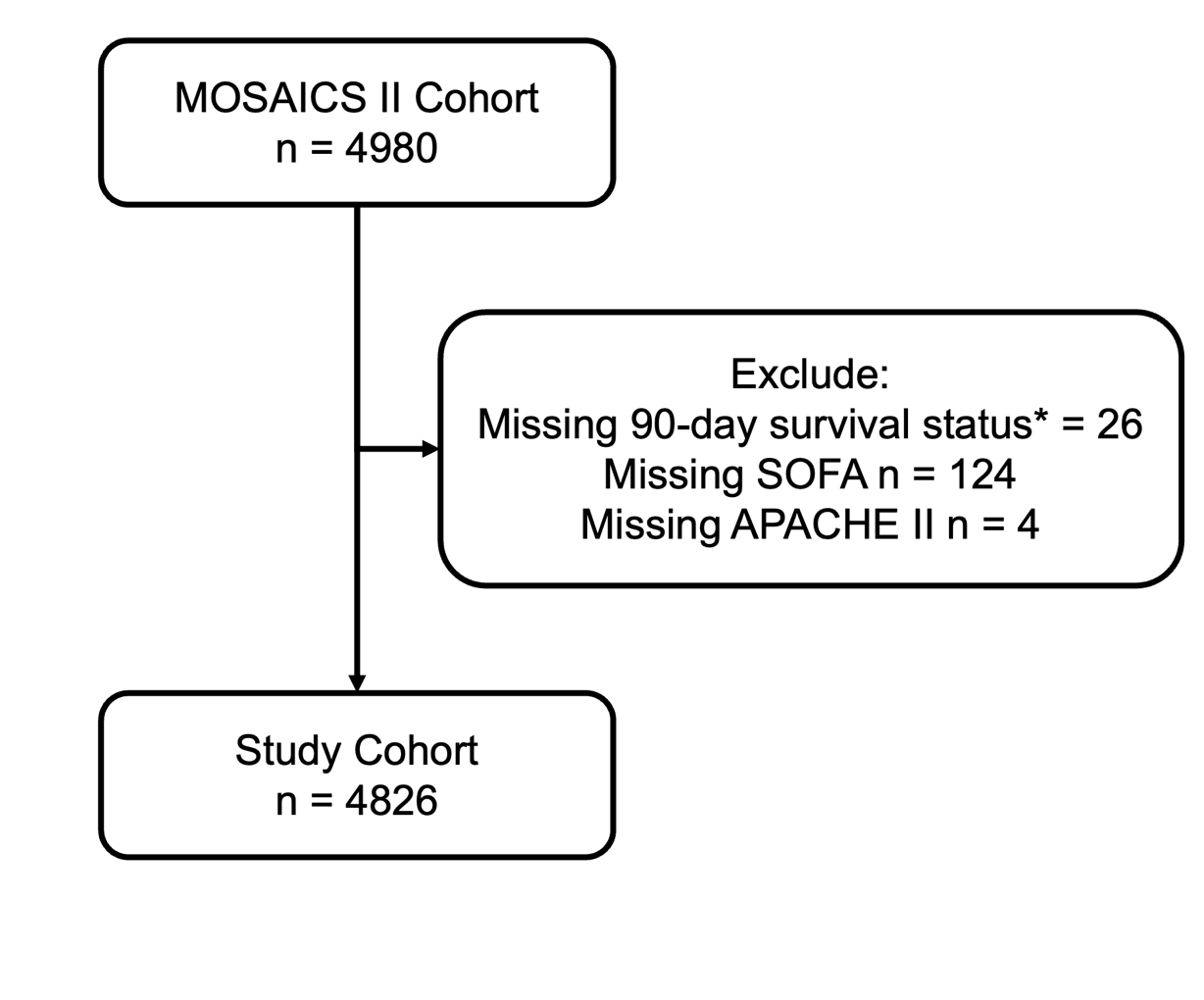
**

*****All patients recruited to MOSAICS II were prospectively followed up for 90 days. A total of 26 patients (9 = low to lower middle income, 9 = upper middle income, 8 = high income countries/regions) had unknown 90-day survival status and was excluded in this secondary analysis. APACHE II, Acute Physiology and Chronic Health Evaluation II; SOFA, Sequential Organ Failure Assessment.

**Supplementary Figure 3: qSOFA components in different income countries/regions
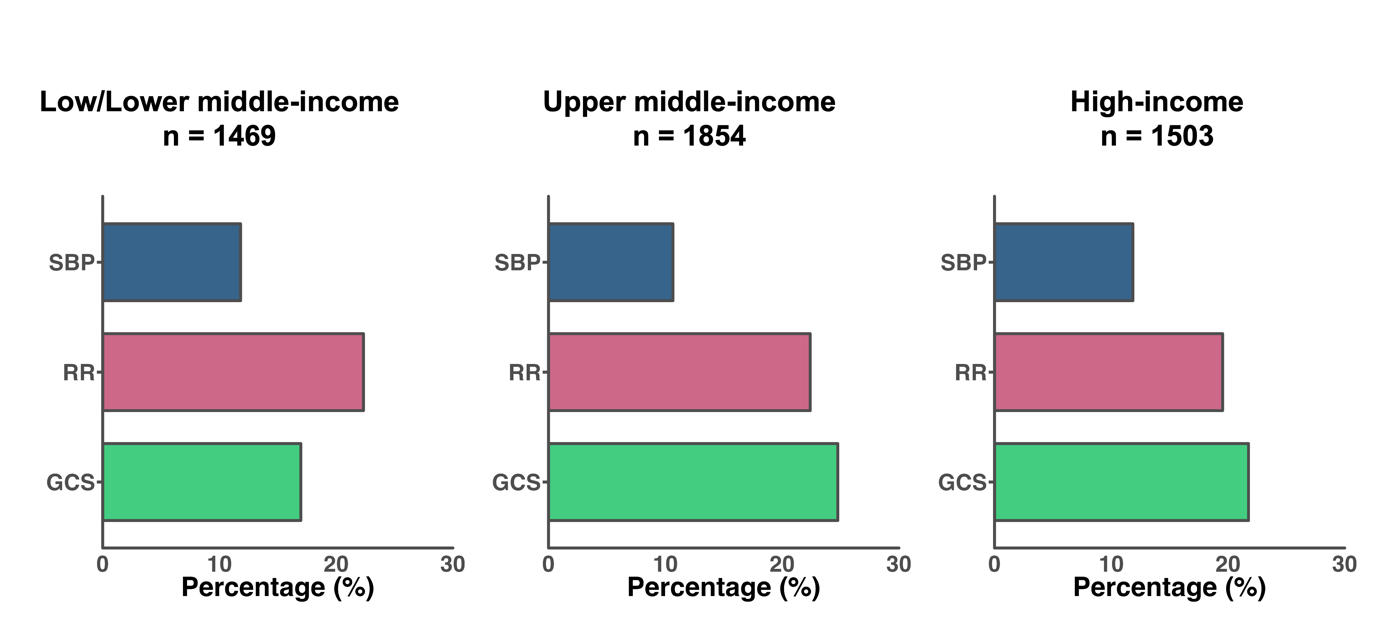
**

Percentage of patients who fulfilled each qSOFA component in each income group. GCS, Glasgow Coma Scale (Altered Mental Status); qSOFA, quick sequential organ failure assessment; RR, respiratory rate; SBP, systolic blood pressure.

**Supplementary Figure 4: Difference in 90-day outcomes between income countries/regions (n = 4826)**

**
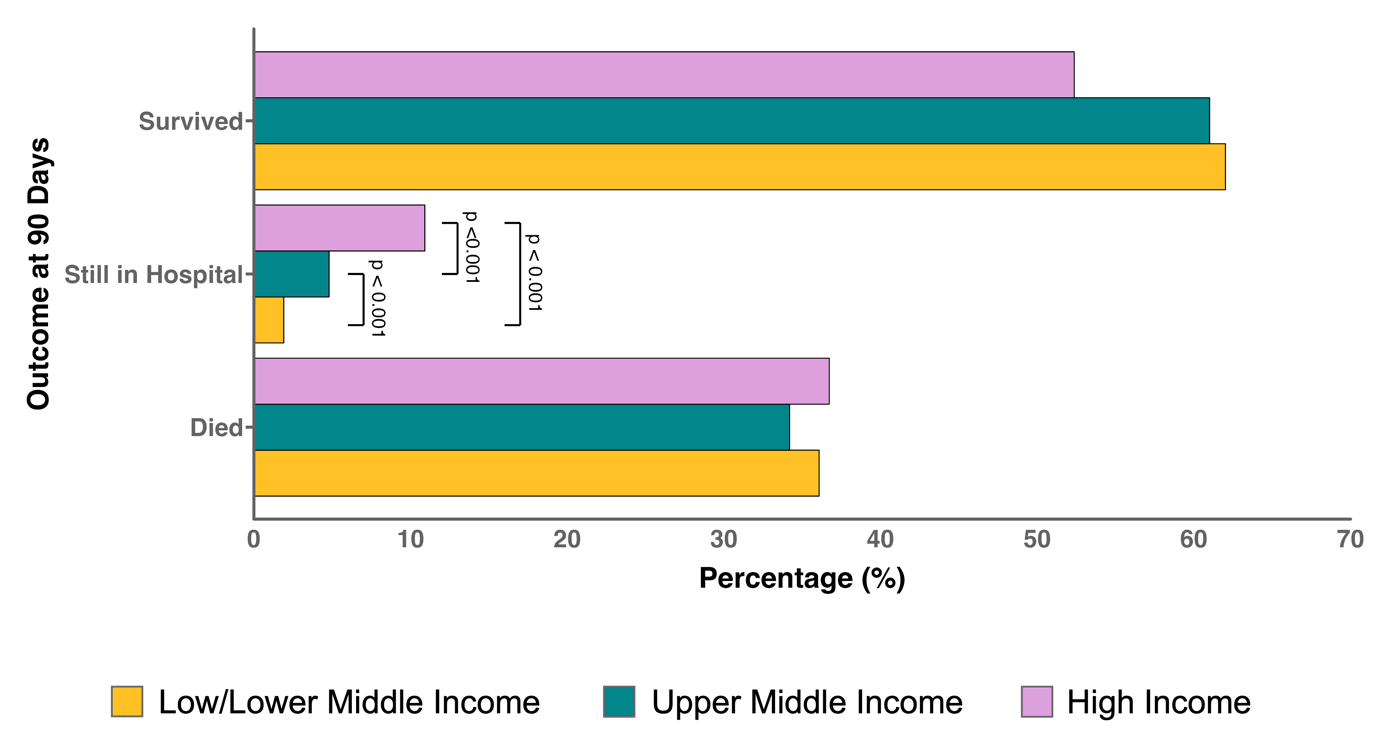
**

Proportion of patients with outcome censored at 90 days in across all country/region income groups.

**Supplementary Figure 5: Difference in 90-day outcomes between income countries/regions in subgroup of patients with lactate measurement (n = 3863)**

**
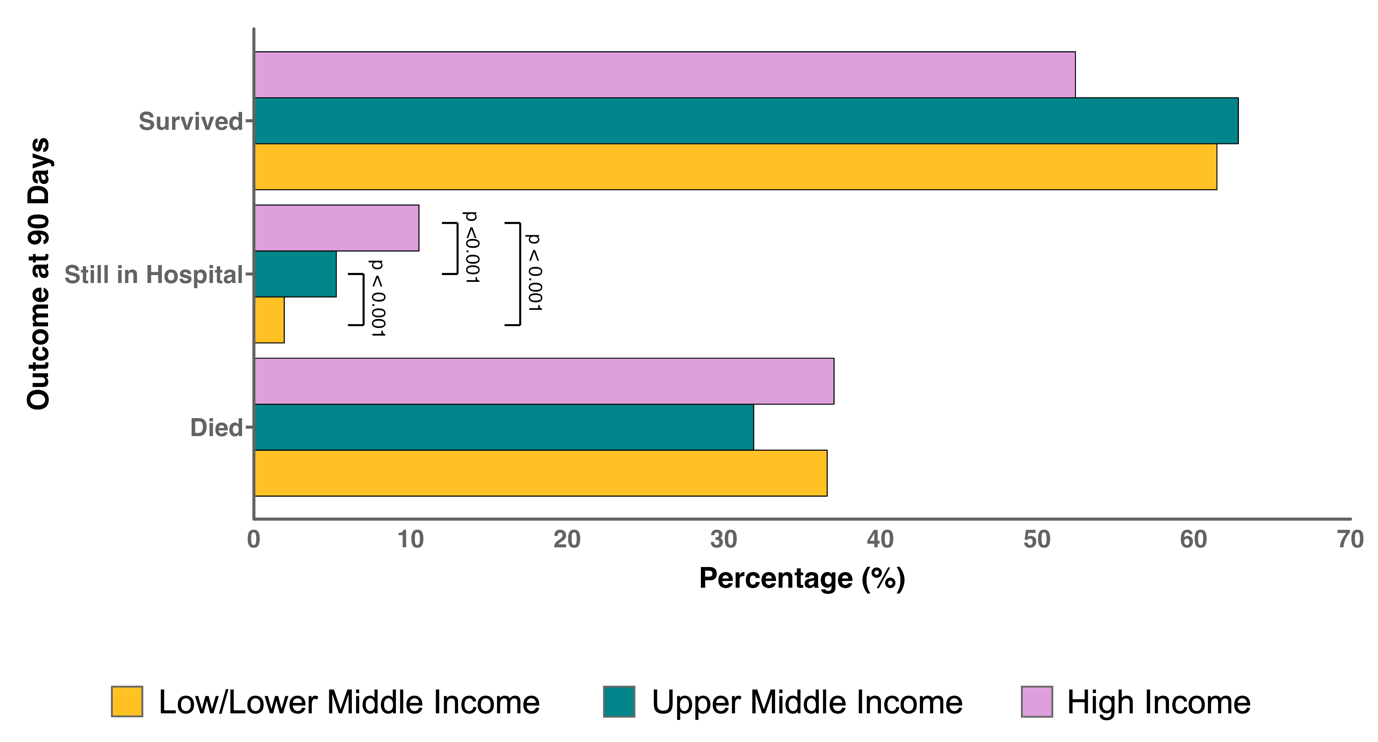
**

Proportion of patients with outcome censored at 90 days in across all country/region income groups.

**Supplementary Figure 6: Discriminatory performance for 28-day mortality of different scores**


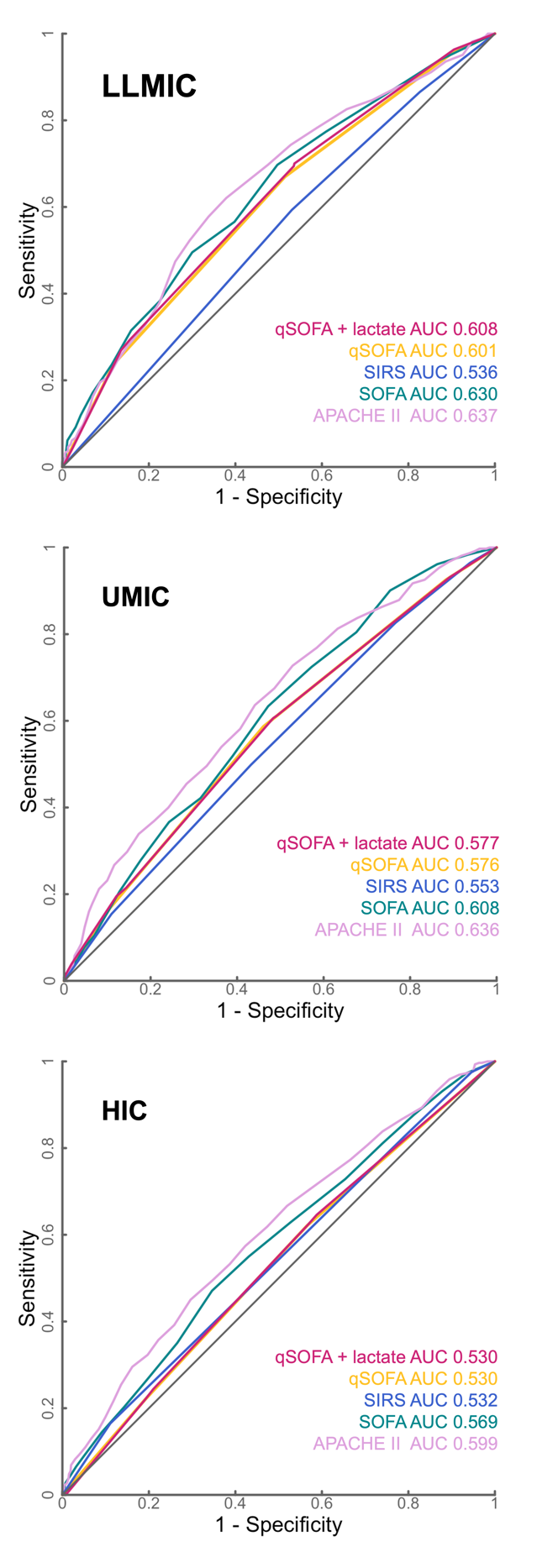


Discriminatory performance of APACHE II, SOFA, SIRS, qSOFA, and qSOFA with lactate for 28-day mortality were evaluated in 3,863 patients after exclusion of 1091 patients who did not have lactate results within 24 hours of ICU admission. APACHE, Acute Physiology And Chronic Health Evaluation; HIC, high income country/region; LLMIC, low and lower middle income country/region; qSOFA, quick sequential organ failure assessment; SOFA, sequential organ failure assessment; UMIC, upper middle income country/region.
