# Supplementary material for: Prognostic evaluation of quick sequential organ failure assessment score in ICU patients with sepsis across different income settings
Source: Crit Care. 2024 Jan 23;28:30. doi: 10.1186/s13054-024-04804-7 (PMC10804657; doi:10.1186/s13054-024-04804-7)
Supplement: Supplementary file 2 — Additional file 2. Supplementary Tables 1–12. [file 13054_2024_4804_MOESM2_ESM.docx]

**Supplementary Tables**

**Supplementary Table 1: qSOFA and mortality in different income countries/regions**

|  | **3-Day Mortality for qSOFA Score** | | | | |
| --- | --- | --- | --- | --- | --- |
|  | **0** | **1** | **2** | **3** | ***p*** |
| **Low to Lower middle income** | 3/109 (2.8) | 22/503 (4.4) | 36/607 (5.9) | 38/250 (15.2) | < 0.001 |
| **Upper middle income** | 1/197 (0.5) | 19/747 (2.5) | 36/687 (5.2) | 20/223 (9.0) | < 0.001 |
| **High income** | 0/93 (0.0) | 6/542 (1.1) | 20/581 (3.4) | 9/287 (3.1) | 0.0164 |

|  | **28-Day Mortality for qSOFA Score** | | | | |
| --- | --- | --- | --- | --- | --- |
|  | **0** | **1** | **2** | **3** | ***p*** |
| **Low to Lower middle income** | 24/109 (22.0) | 129/503 (25.6) | 194/607 (32.0) | 112/250 (44.8) | < 0.001 |
| **Upper middle income** | 31/197 (15.7) | 162/747 (21.7) | 202/687 (29.4) | 79/223 (35.4) | < 0.001 |
| **High income** | 20/93 (21.5) | 106/542 (19.6) | 141/581 (24.3) | 71/287 (24.7) | 0.220 |

|  | **90-Day Mortality for qSOFA Score** | | | | |
| --- | --- | --- | --- | --- | --- |
|  | **0** | **1** | **2** | **3** | ***p*** |
| **Low to Lower middle income** | 30/109 (27.5) | 151/503 (30.0) | 225/607 (37.1) | 124/250 (49.6) | < 0.001 |
| **Upper middle income** | 38/197 (19.3) | 239/747 (32.0) | 260/687 (37.8) | 97/223 (43.5) | < 0.001 |
| **High income** | 30/93 (32.3) | 191/542 (35.2) | 216/581 (37.2) | 115/287 (40.1) | 0.4325 |

Mortality rates on day-3, day-28 and day-90 associated with different qSOFA scores across all income countries/regions.

**Supplementary Table 2: qSOFA components and 28-day mortality in different income countries/regions**

|  | **28-Day Mortality for qSOFA Systolic Blood Pressure** | | |
| --- | --- | --- | --- |
|  | **0** | **1** | ***p*** |
| **Low to Lower middle income** | 249/899 (27.7) | 210/570 (36.8) | < 0.001 |
| **Upper middle income** | 320/1340 (23.9) | 154/514 (30.0) | 0.009 |
| **High income** | 206/931 (22.1) | 132/572 (23.1) | 0.715 |

|  | **28-Day Mortality for qSOFA Respiratory Rate** | | |
| --- | --- | --- | --- |
|  | **0** | **1** | ***p*** |
| **Low to Lower middle income** | 115/391 (29.4) | 344/1078 (31.9) | 0.396 |
| **Upper middle income** | 184/773 (23.8) | 290/1081 (26.8) | 0.156 |
|  |  |  |  |
| **High income** | 113/560 (20.2) | 225/943 (23.9) | 0.112 |

|  | **28-Day Mortality for qSOFA Altered Mental Status** | | |
| --- | --- | --- | --- |
|  | **0** | **1** | ***p*** |
| **Low to Lower middle income** | 160/650 (24.6) | 299/819 (36.5) | < 0.001 |
| **Upper middle income** | 115/659 (17.5) | 359/1195 (30.0) | < 0.001 |
| **High income** | 94/453 (20.8) | 244/1050 (23.2) | 0.321 |

Mortality rates on day-28 associated with different components of qSOFA across all income countries/regions.

**Supplementary Table 3: Multivariate analysis of association between qSOFA and 3-Day Mortality**

|  | **Adjusted Relative Risk** | ***p*** |
| --- | --- | --- |
|  |  |  |
| **Income Region/Country** |  |  |
| High Income | Reference | < 0.001 |
| Upper Middle Income | 1.64 (1.06-2.60) |  |
| Low/Lower Middle Income | 3.77 (2.49-5.85) |  |
| **Age** | 1.00 (0.99-1.01) | 0.705 |
| **Female Sex** | 1.11 (0.81-1.51) | 0.527 |
| **Solid Malignant Tumor** | 1.10 (0.65-1.74) | 0.713 |
| **Immunosuppression** | 0.62 (0.24-1.32) | 0.239 |
| **Hematological Malignancy** | 1.80 (0.92-3.21) | 0.082 |
| **Emergency Department Admission** | 1.25 (0.91-1.72) | 0.174 |
| **Unscheduled Surgical Admission** | 0.78 (0.44-1.31) | 0.366 |
| **Antibiotics within 3 Hours** | 0.52 (0.37-0.74) | <0.001 |
| **APACHE II** | 1.04 (1.02-1.06) | <0.001 |
| **qSOFA** | 1.52 (1.24-1.87) | <0.001 |
| **SOFA** | 1.05 (1.00-1.08) | 0.029 |
|  |  |  |

APACHE, Acute Physiology And Chronic Health Evaluation; qSOFA, quick sequential organ failure assessment; SOFA, sequential organ failure assessment.

**Supplementary Table 4 Multivariate analysis of association between qSOFA and 90-Day Mortality**

|  | **Adjusted Relative Risk** | ***p*** |
| --- | --- | --- |
|  |  |  |
| **Income Region/Country** |  |  |
| High Income | Reference | < 0.001 |
| Upper Middle Income | 0.98 (0.86-1.12) |  |
| Low/Lower Middle Income | 1.26 (1.10-1.45) |  |
| **Age** | 1.00 (1.00-1.01) | 0.080 |
| **Female Sex** | 0.93 (0.84-1.04) | 0.224 |
| **Solid Malignant Tumor** | 1.35 (1.17-1.56) | <0.001 |
| **Immunosuppression** | 1.25 (1.00-1.56) | 0.051 |
| **Hematological Malignancy** | 1.27 (0.99-1.61) | 0.058 |
| **Emergency Department Admission** | 0.91 (0.82-1.01) | 0.086 |
| **Unscheduled Surgical Admission** | 0.89 (0.75-1.05) | 0.156 |
| **Antibiotics within 3 Hours** | 0.61 (0.55-0.69) | <0.001 |
| **APACHE II** | 1.02 (1.02-1.03) | <0.001 |
| **qSOFA** | 1.03 (0.96-1.100) | 0.454 |
| **SOFA** | 1.03 (1.01-1.04) | <0.001 |
|  |  |  |

APACHE, Acute Physiology And Chronic Health Evaluation; qSOFA, quick sequential organ failure assessment; SOFA, sequential organ failure assessment.

**Supplementary Table 5: Predictive performance of different scores in overall cohort**

| **Score** | **Mortality Day** | **Thresholds** | **AUC (95%CI)** | **Sensitivity (95%CI)** | **Specificity (95%CI)** |
| --- | --- | --- | --- | --- | --- |
| qSOFA+  lactate | 3 | 0.04 | 0.644 (0.602-0.685) | 0.736 (0.667-0.804) | 0.490 (0.474-0.506) |
| qSOFA | 3 | 1.5 | 0.642 (0.601-0.682) | 0.748 (0.681-0.816) | 0.469 (0.453-0.486) |
| SIRS | 3 | 1.5 | 0.586 (0.548-0.625) | 0.937 (0.899-0.975) | 0.203 (0.190-0.216) |
| SOFA | 3 | 7.5 | 0.605 (0.560-0.650) | 0.635 (0.560-0.710) | 0.521 (0.505-0.537) |
| APACHE II | 3 | 19.5 | 0.659 (0.618-0.700) | 0.742 (0.674-0.810) | 0.485 (0.469-0.501) |
| qSOFA+lactate | 28 | 0.262 | 0.572 (0.552-0.592) | 0.625 (0.595-0.655) | 0.490 (0.471-0.508) |
| qSOFA | 28 | 1.5 | 0.570 (0.551-0.589) | 0.625 (0.595-0.655) | 0.490 (0.471-0.508) |
| SIRS | 28 | 2.5 | 0.547 (0.527-0.566) | 0.527 (0.496-0.558) | 0.537 (0.519-0.556) |
| SOFA | 28 | 7.5 | 0.590 (0.570-0.610) | 0.586 (0.555-0.617) | 0.549 (0.531-0.567) |
| APACHE II | 28 | 20.5 | 0.612 (0.592-0.632) | 0.608 (0.577-0.638) | 0.566 (0.547-0.584) |
| qSOFA+lactate | 90 | 0.346 | 0.563 (0.545-0.581) | 0.621 (0.595-0.647) | 0.468 (0.449-0.488) |
| qSOFA | 90 | 1.5 | 0.559 (0.541-0.577) | 0.595 (0.569-0.621) | 0.490 (0.471-0.510) |
| SIRS | 90 | 3.5 | 0.528 (0.510-0.546) | 0.167 (0.147-0.187) | 0.872 (0.859-0.885) |
| SOFA | 90 | 7.5 | 0.598 (0.580-0.617) | 0.586 (0.560-0.612) | 0.569 (0.549-0.588) |
| APACHE II | 90 | 20.5 | 0.629 (0.610-0.647) | 0.607 (0.581-0.633) | 0.590 (0.571-0.610) |

Analysis in overall cohort (n = 3863) after exclusion of patients without lactate results within 24 hours of ICU admission. Thresholds were selected based on Youden’s index. APACHE, Acute Physiology And Chronic Health Evaluation; AUC, area under the curve; qSOFA, quick sequential organ failure assessment; SOFA, sequential organ failure assessment.

**Supplementary Table 6: Predictive performance of different scores in low/low middle income countries/regions**

| **Score** | **Mortality Day** | **Thresholds** | **AUC (95%CI)** | **Sensitivity (95%CI)** | **Specificity (95%CI)** |
| --- | --- | --- | --- | --- | --- |
| qSOFA+lactate | 3 | 0.098 | 0.659 (0.593-0.726) | 0.397 (0.285-0.510) | 0.860 (0.838-0.882) |
| qSOFA | 3 | 2.5 | 0.658 (0.592-0.724) | 0.397 (0.285-0.510) | 0.860 (0.838-0.882) |
| SIRS | 3 | 2.5 | 0.593 (0.536-0.649) | 0.699 (0.593-0.804) | 0.464 (0.432-0.495) |
| SOFA | 3 | 7.5 | 0.658 (0.590-0.726) | 0.603 (0.490-0.715) | 0.657 (0.627-0.687) |
| APACHE II | 3 | 17.5 | 0.699 (0.641-0.758) | 0.808 (0.718-0.899) | 0.522 (0.490-0.553) |
| qSOFA+lactate | 28 | 0.249 | 0.608 (0.573-0.643) | 0.700 (0.651-0.750) | 0.456 (0.419-0.493) |
| qSOFA | 28 | 1.5 | 0.601 (0.566-0.636) | 0.670 (0.619-0.721) | 0.477 (0.440-0.514) |
| SIRS | 28 | 2.5 | 0.536 (0.500-0.572) | 0.593 (0.540-0.647) | 0.473 (0.436-0.510) |
| SOFA | 28 | 7.5 | 0.630 (0.593-0.666) | 0.495 (0.441-0.550) | 0.701 (0.667-0.735) |
| APACHE II | 28 | 18.5 | 0.637 (0.601-0.674) | 0.621 (0.568-0.673) | 0.615 (0.579-0.651) |
| qSOFA+lactate | 90 | 0.315 | 0.600 (0.566-0.634) | 0.685 (0.638-0.732) | 0.460 (0.421-0.498) |
| qSOFA | 90 | 1.5 | 0.591 (0.557-0.625)* | 0.653 (0.605-0.701) | 0.479 (0.441-0.518) |
| SIRS | 90 | 2.5 | 0.511 (0.476-0.547) | 0.566 (0.516-0.616) | 0.463 (0.424-0.501) |
| SOFA | 90 | 7.5 | 0.637 (0.602-0.673) | 0.492 (0.442-0.542) | 0.715 (0.680-0.749) |
| APACHE II | 90 | 19.5 | 0.649 (0.614-0.683)* | 0.574 (0.524-0.624) | 0.672 (0.636-0.708) |

^*^Pair wise comparison between qSOFA and APACHE II using Delong’s test (*p* = 0.003)

Analysis in LMIC cohort (n = 1469) after exclusion of patients without lactate results within 24 hours of ICU admission. Thresholds were selected based on Youden’s index. APACHE, Acute Physiology And Chronic Health Evaluation; AUC, area under the curve; qSOFA, quick sequential organ failure assessment; SOFA, sequential organ failure assessment.

**Supplementary Table 7: Predictive performance of different scores in upper middle income countries/regions**

| **Score** | **Mortality Day** | **Thresholds** | **AUC** | **Sensitivity** | **Specificity** |
| --- | --- | --- | --- | --- | --- |
| qSOFA+lactate | 3 | 0.035 | 0.635 (0.565-0.705) | 0.673 (0.546-0.801) | 0.540 (0.515-0.565) |
| qSOFA | 3 | 1.5 | 0.633 (0.565-0.702) | 0.692 (0.567-0.818) | 0.519 (0.494-0.544) |
| SIRS | 3 | 1.5 | 0.571 (0.505-0.638) | 0.923 (0.851-0.996) | 0.228 (0.207-0.250) |
| SOFA | 3 | 7.5 | 0.595 (0.520-0.669) | 0.654 (0.525-0.783) | 0.497 (0.471-0.522) |
| APACHE II | 3 | 23.5 | 0.661 (0.595-0.726) | 0.615 (0.483-0.748) | 0.641 (0.617-0.665) |
| qSOFA+lactate | 28 | 0.246 | 0.577 (0.545-0.609) | 0.587 (0.536-0.637) | 0.542 (0.514-0.571) |
| qSOFA | 28 | 1.5 | 0.576 (0.545-0.608) | 0.587 (0.536-0.637) | 0.542 (0.514-0.571) |
| SIRS | 28 | 2.5 | 0.553 (0.521-0.585) | 0.499 (0.447-0.550) | 0.572 (0.544-0.600) |
| SOFA | 28 | 7.5 | 0.608 (0.576-0.640) | 0.634 (0.584-0.683) | 0.530 (0.501-0.558) |
| APACHE II | 28 | 18.5 | 0.636 (0.604-0.669) | 0.727 (0.681-0.773) | 0.473 (0.444-0.501) |
| qSOFA+lactate | 90 | 0.328 | 0.568 (0.539-0.597) | 0.577 (0.534-0.621) | 0.519 (0.489-0.549) |
| qSOFA | 90 | 1.5 | 0.563 (0.535-0.592) | 0.549 (0.506-0.593) | 0.541 (0.511-0.571) |
| SIRS | 90 | 3.5 | 0.538 (0.508-0.568) | 0.157 (0.125-0.189) | 0.899 (0.881-0.917) |
| SOFA | 90 | 7.5 | 0.608 (0.579-0.637) | 0.626 (0.583-0.668) | 0.547 (0.517-0.577) |
| APACHE II | 90 | 20.5 | 0.645 (0.616-0.674) | 0.628 (0.585-0.670) | 0.582 (0.552-0.611) |

Analysis in UMIC cohort (n = 1854) after exclusion of patients without lactate results within 24 hours of ICU admission. Thresholds were selected based on Youden’s index. APACHE, Acute Physiology And Chronic Health Evaluation; AUC, area under the curve; qSOFA, quick sequential organ failure assessment; SOFA, sequential organ failure assessment.

**Supplementary Table 8: Predictive performance of different scores in high income countries/regions**

| **Score** | **Mortality Day** | **Thresholds** | **AUC** | **Sensitivity** | **Specificity** |
| --- | --- | --- | --- | --- | --- |
| qSOFA+lactate | 3 | 0.027 | 0.637 (0.559-0.714) | 0.824 (0.695-0.952) | 0.458 (0.430-0.486) |
| qSOFA | 3 | 1.5 | 0.626 (0.551-0.701) | 0.824 (0.695-0.952) | 0.428 (0.401-0.456) |
| SIRS | 3 | 1.5 | 0.538 (0.453-0.624) | 0.912 (0.816-1.000) | 0.199 (0.176-0.221) |
| SOFA | 3 | 10.5 | 0.623 (0.529-0.718) | 0.471 (0.303-0.638) | 0.713 (0.688-0.738) |
| APACHE II | 3 | 30.5 | 0.707 (0.608-0.806) | 0.559 (0.392-0.726) | 0.844 (0.824-0.864) |
| qSOFA+lactate | 28 | 0.218 | 0.530 (0.494-0.566) | 0.622 (0.566-0.678) | 0.434 (0.403-0.465) |
| qSOFA | 28 | 1.5 | 0.530 (0.495-0.566) | 0.622 (0.566-0.678) | 0.434 (0.403-0.465) |
| SIRS | 28 | 3.5 | 0.532 (0.496-0.568) | 0.165 (0.122-0.208) | 0.885 (0.865-0.905) |
| SOFA | 28 | 9.5 | 0.569 (0.532-0.606) | 0.471 (0.413-0.528) | 0.645 (0.615-0.675) |
| APACHE II | 28 | 20.5 | 0.599 (0.562-0.636) | 0.667 (0.613-0.721) | 0.476 (0.445-0.507) |
| qSOFA+lactate | 90 | 0.374 | 0.522 (0.491-0.554) | 0.597 (0.552-0.641) | 0.432 (0.398-0.466) |
| qSOFA | 90 | 1.5 | 0.522 (0.491-0.553) | 0.597 (0.552-0.641) | 0.432 (0.398-0.466) |
| SIRS | 90 | 3.5 | 0.524 (0.492-0.555) | 0.151 (0.118-0.183) | 0.888 (0.866-0.910) |
| SOFA | 90 | 9.5 | 0.564 (0.531-0.597) | 0.452 (0.407-0.497) | 0.660 (0.628-0.693) |
| APACHE II | 90 | 25.5 | 0.601 (0.569-0.633) | 0.435 (0.390-0.480) | 0.714 (0.683-0.745) |

Analysis in HIC cohort (n = 1503) after exclusion of patients without lactate results within 24 hours of ICU admission. Thresholds were selected based on Youden’s index. APACHE, Acute Physiology And Chronic Health Evaluation; AUC, area under the curve; qSOFA, quick sequential organ failure assessment; SOFA, sequential organ failure

**Supplementary Table 9: Predictive performance of different scores for hospital mortality in overall cohort**

| **Score** | **Mortality** | **Thresholds** | **AUC** | **Sensitivity** | **Specificity** |
| --- | --- | --- | --- | --- | --- |
| qSOFA+lactate | Hospital | 0.382 | 0.568 (0.550-0.586) | 0.621 (0.595-0.647) | 0.473 (0.453-0.494) |
| qSOFA | Hospital | 1.5 | 0.561 (0.543-0.579)* | 0.595 (0.569-0.621) | 0.492 (0.472-0.513) |
| SIRS | Hospital | 3.5 | 0.528 (0.509-0.546) | 0.167 (0.147-0.187) | 0.871 (0.857-0.885) |
| SOFA | Hospital | 7.5 | 0.604 (0.585-0.623) | 0.586 (0.560-0.612) | 0.577 (0.557-0.598) |
| APACHE II | Hospital | 20.5 | 0.636 (0.618-0.655)* | 0.607 (0.581-0.633) | 0.600 (0.580-0.620) |

^*^Pair wise comparison between qSOFA and APACHE II using Delong’s test (*p* < 0.001)

Subgroup analysis of overall cohort (n = 3627) after exclusion of patients who remained in hospital by day 90. Thresholds were selected based on Youden’s index. APACHE, Acute Physiology And Chronic Health Evaluation; AUC, area under the curve; qSOFA, quick sequential organ failure assessment; SOFA, sequential organ failure assessment.

**Supplementary Table 10: Predictive performance of different scores for hospital mortality in low/low middle income countries/regions**

| **Score** | **Mortality** | **Thresholds** | **AUC** | **Sensitivity** | **Specificity** |
| --- | --- | --- | --- | --- | --- |
| qSOFA+lactate | Hospital | 0.319 | 0.603 (0.569-0.638) | 0.685 (0.638-0.732) | 0.468 (0.429-0.507) |
| qSOFA | Hospital | 1.5 | 0.594 (0.560-0.628)* | 0.653 (0.605-0.701) | 0.488 (0.449-0.527) |
| SIRS | Hospital | 2.5 | 0.510 (0.475-0.545) | 0.566 (0.516-0.616) | 0.458 (0.420-0.497) |
| SOFA | Hospital | 7.5 | 0.639 (0.604-0.674) | 0.492 (0.442-0.542) | 0.713 (0.678-0.749) |
| APACHE II | Hospital | 19.5 | 0.654 (0.619-0.689)* | 0.574 (0.524-0.624) | 0.679 (0.642-0.715) |

^*^Pair wise comparison between qSOFA and APACHE II using Delong’s test (*p* = 0.003)

Subgroup analysis of LMIC cohort (n = 1013) after exclusion of patients who remained in hospital by day 90. Thresholds were selected based on Youden’s index. APACHE, Acute Physiology And Chronic Health Evaluation; AUC, area under the curve; qSOFA, quick sequential organ failure assessment; SOFA, sequential organ failure assessment.

**Supplementary Table 11: Predictive performance of different scores for hospital mortality in upper middle income countries/regions**

| **Score** | **Mortality** | **Thresholds** | **AUC** | **Sensitivity** | **Specificity** |
| --- | --- | --- | --- | --- | --- |
| qSOFA+lactate | Hospital | 0.327 | 0.569 (0.539-0.598) | 0.581 (0.538-0.625) | 0.510 (0.478-0.541) |
| qSOFA | Hospital | 1.5 | 0.562 (0.533-0.591)* | 0.549 (0.506-0.593) | 0.537 (0.506-0.569) |
| SIRS | Hospital | 3.5 | 0.534 (0.504-0.564) | 0.157 (0.125-0.189) | 0.898 (0.879-0.917) |
| SOFA | Hospital | 7.5 | 0.608 (0.578-0.637) | 0.626 (0.583-0.668) | 0.546 (0.515-0.578) |
| APACHE II | Hospital | 20.5 | 0.646 (0.617-0.675)* | 0.628 (0.585-0.670) | 0.581 (0.550-0.612) |

^*^Pair wise comparison between qSOFA and APACHE II using Delong’s test (*p* < 0.001)

Subgroup analysis of UMIC cohort (n = 1476) after exclusion of patients who remained in hospital by day 90. Thresholds were selected based on Youden’s index. APACHE, Acute Physiology And Chronic Health Evaluation; AUC, area under the curve; qSOFA, quick sequential organ failure assessment; SOFA, sequential organ failure assessment.

**Supplementary Table 12: Predictive performance of different scores for hospital mortality in high income countries/regions**

| **Score** | **Mortality** | **Thresholds** | **AUC** | **Sensitivity** | **Specificity** |
| --- | --- | --- | --- | --- | --- |
| qSOFA+lactate | Hospital | 0.422 | 0.525 (0.493-0.558) | 0.263 (0.223-0.303) | 0.769 (0.737-0.801) |
| qSOFA | Hospital | 2.5 | 0.522 (0.490-0.554)* | 0.214 (0.177-0.252) | 0.813 (0.783-0.842) |
| SIRS | Hospital | 1.5 | 0.533 (0.501-0.566) | 0.828 (0.794-0.862) | 0.217 (0.186-0.249) |
| SOFA | Hospital | 9.5 | 0.573 (0.539-0.606) | 0.452 (0.407-0.497) | 0.675 (0.639-0.710) |
| APACHE II | Hospital | 25.5 | 0.611 (0.578-0.644)* | 0.435 (0.390-0.480) | 0.735 (0.701-0.768) |

^*^Pair wise comparison between qSOFA and APACHE II using Delong’s test (*p* < 0.001)

Subgroup analysis of HIC cohort (n = 1138) after exclusion of patients who remained in hospital by day 90. Thresholds were selected based on Youden’s index. APACHE, Acute Physiology And Chronic Health Evaluation; AUC, area under the curve; qSOFA, quick sequential organ failure assessment; SOFA, sequential organ failure assessment.
