# Supplementary material for: Prognostic evaluation of quick sequential organ failure assessment score in ICU patients with sepsis across different income settings
Source: Crit Care. 2024 Jan 23;28:30. doi: 10.1186/s13054-024-04804-7 (PMC10804657; doi:10.1186/s13054-024-04804-7)
Supplement: Supplementary file 3 — Additional file 3. Appendix 1: Contributing centers listed alphabetically per country/region with Lead Investigators and Co-Investigators. [file 13054_2024_4804_MOESM3_ESM.docx]

**MOSAICS II Study Group**

**Appendix 1:** **Contributing centers listed alphabetically per country/region with Lead Investigators and Co-Investigators**

**Bangladesh**

| **Site number** | **Hospital name** | **Investigator/Co-investigator** |
| --- | --- | --- |
| 1 | Anwar Khan Modern Hospital | Uzzal Kumar Mallick |
| 2 | Asgar Ali Hospital | Motiul Islam |
| 3 | Central Hospital | Tarequl Hamid |
| 4 | CKD & Urology Hospital | A KM Shirazul Islam |
| 5 | Dhanmondi Clinic | Rabiul Halim |
| 6 | Eden Multi Care Hospital | Md Arifur Rahman Khan |
| 7 | Green Life Medical College & Hospital | Mohammad Asaduzzaman |
| 8 | Ibn Sina Specialized Hospital, Dhanmondi | Md Rezaul Karim |
| 9 | Japan Bangladesh friendship Hospital | Nahim Sarwar |
| 10 | National Institute of Neurosciences & Hospital (NINS) | Shamsul Hoque Milon |
| 11 | Oasis Medical College Hospital | Rashed Mahmud |
| 12 | Sarkari Karmachari Hospital | A K M Sirajul Islam Hirok |
| 13 | Sheikh Fazilatunnesa Mujib Memorial KPJ Specialized Hospital | Ashraful Haque |
| 14 | Unied Hospital Ltd | Amina Sultana / Mir Atiqur Rahman Shajal |
| 15 | Universal Medical College & Hospital Ltd | Farha Andalib |
| 16 | Uttara Cresent Hospital | Rashedul Hasan |

**Brunei**

| **Site number** | **Hospital name** | **Investigator/Co-investigator** |
| --- | --- | --- |
| 17 | RIPAS | Khalid Mahmood Khan Nafees |
| 18 | Suri Seri Begawan Hospital Kuala Belait Brunei | Shah Sudhirchandra Dhansukhlal |

**China**

| **Site number** | **Hospital name** | **Investigator/Co-investigator** |
| --- | --- | --- |
| 19 | Affiliated Hospital of Hebei University | Ning Li |
| 20 | Affiliated Hospital of Jining Medical University | Xiaowei Liu / Haiwei Yang |
| 21 | Affiliated Hospital of Qinghai University | Ming Hou |
| 22 | Baoding General Hospital | Ying Li / Jian Zhang |
| 23 | Beijing Chaoyang Hospital | Lifeng Huang / Wenxiong Li |
| 24 | Beijing Friendship Hospital, Capital Medical University | Meili Duan |
| 25 | Beijing Hospital | Taotao Liu |
| 26 | Beijing Tongren Hospital, CMU | Wei He |
| 27 | Binzhou Medical University Hospital | Fangyu Ning / Xiaozhi Wang |
| 28 | Cangzhou Hospital of Integrated TCM-WM, Hebei | Xiaoyan Zhou |
| 29 | Changshu No.1 People's Hospital | Sun Yu |
| 30 | Chengdu Fifth People's hospital | Xiang Xiang |
| 31 | Chinese PLA General Hospital | Liang Pan / Feihu Zhou |
| 32 | Chongqing Daping Hospital | Yaoli Wang / Jian Zhou |
| 33 | Chongqing Emergency Medical Center | Tao Wang / Xuefei Yang / Yu Ma |
| 34 | Dong'e General Hospital | Xuan Song |
| 35 | First Affiliated Hospital of Kunming Medical University | Haiying Wu / Chuanyun Qian |
| 36 | Foshan No.1 People's Hospital | Lixin Zhou / Zuohang Xu |
| 37 | Fourth Hospital of Hebei Medical University | Kun Zhang / Zhenjie Hu |
| 38 | Fujian Provincial Hospital (Medical ICU) | Xingsheng Lin / Songjing Shi |
| 39 | Fujian Provincial Hospital (Surgical ICU) | Xiaoguang Zhang / Rongguo Yu |
| 40 | Gansu Provincial Hospital (ICU 1) | Liqin Zhang / Yuan Yuan |
| 41 | Gansu Provincial Hospital (ICU 2) | Huiru Zhou / Xiandong Wang |
| 42 | Guangdong General Hospital | Zhonghua Wang / Tiehe Qin |
| 43 | Guizhou Provincial People's Hospital | Xianqing Shi |
| 44 | Hainan General Hospital | Rui Li / Zhenyang He |
| 45 | Jiangsu Province Hospital | Xiangrong Zuo / Quan Cao |
| 46 | Jiaxing No.2 Hospital | Tao He |
| 47 | Liaocheng General Hospital | Yuanda Sui / Tiejun Wu |
| 48 | Nanjing Drum Tower Hospital | Ying Xu / Qin Gu |
| 49 | Navy General Hospital | Weizheng Shuai |
| 50 | Peking Union Medical College Hospital | Hanyu Qin / Bin Du |
| 51 | Peking University First Hospital | Hong Qiao /Shuangling Li |
| 52 | Peking University People's Hospital | Guiying Dong |
| 53 | Peking University People's Hospital | Xiujuan Zhao / Fengxue Zhu |
| 54 | Peking University Shenzhen Hospital | Junshi Wang /Lei Huang |
| 55 | Qian Xi Nan People's Hospital | Tianchang Wang |
| 56 | Qinghai Provincial People's Hospital | Hao Wang / Siqing Ma / Zhengping Yang |
| 57 | Renji Hospital Shanghai Jiaotong University School of Medicine | Yuan Gao |
| 58 | Ruijin Hospital | Ruoming Tan |
| 59 | Shanghai General Hospital | Yun Xie / Ruilan Wang |
| 60 | Shengjing Hospital of China Medical University | Jia Jia / Bin Zang |
| 61 | Sichuan No.2 Hospital of TCM | Jun Wang |
| 62 | Sir Run Run Shaw Hospital | Ling Lin |
| 63 | Suzhou Municipal Hospital (East Area) | Yuwen Wu / Yunfu Wu |
| 64 | The 8th Medical Center, General Hospital of Chinese People's Liberation Army | Penglin Ma |
| 65 | The Central Hospital of Wuhan | Yanfang Li / Li Yu |
| 66 | The First Affiliated Hospital of Chongqing Medical University | Rui Guo |
| 67 | The First Affiliated Hospital of Dalian Medical University | Jiuzhi Zhang / Xianyao Wan |
| 68 | The First Affiliated Hospital of Guizhou Medical University | Feng Shen |
| 69 | The First Affiliated Hospital of Xi'an Jiaotong University | Qindong Shi |
| 70 | The First Affiliated Hospital of Zhejiang University | Jun Xu / Qiang Fang |
| 71 | The First Affiliated Hospital of Zhengzhou University | Shaohua Liu / Tongwen Sun |
| 72 | The First Affiliated Hospital, Sun Yat-sen University | Mian Zeng |
| 73 | The First Bethune Hospital of Jilin University | Weiyun Pan / Zhongmin Liu |
| 74 | The First Hospital of Lanzhou University | Qingling Lin |
| 75 | The Fourth Hospital of Anhui Medical University | Nan Wang |
| 76 | The People's Hospital of Guangxi Zhuang Autonomous Region | Jing Pang / Bin Xiong |
| 77 | The Second Affiliated Hospital of Guangzhou Medical University | Deliang Wen |
| 78 | The Second Affiliated Hospital of Hainan Medical University | Fuxin Kang |
| 79 | The Second Affiliated Hospital of Soochow University | Liuhui Chang |
| 80 | The Second Hospital of Anhui Medical University | Yun Sun |
| 81 | The Second Hospital of Jilin University | Jingxiao Zhang / Yongjie Yin |
| 82 | The Second People's Hospital of Liaocheng | Liu Qing / Jiajun Sun |
| 83 | Tianjin First Center Hospital | Nahui Li / Yongqiang Wang |
| 84 | Tianjin Medical University General Hospital | Songtao Shou / Yanfen Chai |
| 85 | Tianjin Third Central Hospital | Lei Xu |
| 86 | Union Hospital, Tongji Medical College, Huazhong University of Science and Technology | Xiaobo Yang |
| 87 | West China Hospital, Sichuan University | Xuelian Liao / Xian Kang |
| 88 | Xiangya Hospital Central South University | Shuangping Zhao |
| 89 | Zhejiang Provincial Hospital of TCM | Liquan Huang |
| 90 | Zhejiang Provincial People's Hospital | Run Zhang / Renhua Sun |
| 91 | Zhuhai People's Hospital | Chao Shen / Yan He |

**Hong Kong SAR, China**

| **Site number** | **Hospital name** | **Investigator/Co-investigator** |
| --- | --- | --- |
| 92 | Caritas Medical Centre | Fu Loi Chow / Michele Tang |
| 93 | North District Hospital | Philip Lam / Esther Cham |
| 94 | Pamela Youde Nethersole Eastern Hospital | Kin Bong Tang |
| 95 | Prince of Wales Hospital | Lowell Ling |
| 96 | Princess Margaret Hospital | Manimala Dharmangadan |
| 97 | Queen Mary Hospital | Pauline Yeung Ng |
| 98 | Tuen Mun Hospital | Kin Ho Ling |
| 99 | Yan Chai Hospital | Vincent Lau |

**India**

| **Site number** | **Hospital name** | **Investigator/Co-investigator** |
| --- | --- | --- |
| 100 | AMRI Hospitals | Samir Sahu |
| 101 | AMRI Hospitals, Dhakuria | Sharmila Chatterjee |
| 102 | AMRI Hospitals, Mukundapur | Sushmita Basu |
| 103 | Amrita Institute of Medical Sciences | Zubair Umer Mohamed |
| 104 | Apollo Health City Advanced Critical Care Unit (ACCU) | Sudeep Sirga |
| 105 | Apollo Health City Medical ICU 1 | Siddhartha Reddy Kasireddy |
| 106 | Apollo Health City Medical ICU 2 | M A Aleem |
| 107 | Apollo Health City Surgical ICU | Swarna Deepak Kuragayala |
| 108 | Apollo Health City Tele ICU | Sai Praveen Haranath |
| 109 | Apollo Hospital Chennai | Nagarajan Ramakrishnan |
| 110 | Bombay Hospital Institute of Medical Sciences | Pravin Amin |
| 111 | Breach Candy Hospital Trust | Joanne Mascarenhas |
| 112 | Cancer Institute (WIA) | Radhika Dash |
| 113 | Care Hospital Banjara Hills | Venkat Raman Kola |
| 114 | Cauvery Heart and Multispeciality Hospital | Vaidyanathan R |
| 115 | Charak Icu Siddharth Hospital | Siddharth Agarwal |
| 116 | Chirayu Medical College and Hospital | Pradip K Bhattacharya |
| 117 | Criticare Hospital & Research Institute | Deepak Jeswani |
| 118 | Dayanand Medical College and Hospital | Parshotum Lal Gautam |
| 119 | Dr Balabhai Nanavati Hospital | Abdul Samad Ansari |
| 120 | Fortis Flt Lt Rajan Dhall Hospital, Vasant Kunj. | Vivek Nangia |
| 121 | Fortis Hospital Noida | Mrinal Sircar |
| 122 | Ganga Medical Centre & Hospitals | V.M.Balasubramani |
| 123 | Gleneagles Global Hospital | S. Maneendra |
| 124 | Ims & Sum Hospital | Sanghamitra Mishra |
| 125 | Indira Gandhi Institute Of Medical Sciences | Anjeev Kumar |
| 126 | Indraprastha Apollo Hospitals | Rajesh Chawla |
| 127 | Kasturba Medical College Manipal University | Trevor Francis Sequeira |
| 128 | Kothari Medical and Research Institute | Om Prakash Shrivastava |
| 129 | Lisie Hospital | Sreevalsan TV |
| 130 | Manipal Hospital Whitefield | Rajesh Mohan Shetty / Manjunath Thimmappa |
| 131 | Mazmdar Shaw Medical Centre Narayana Hrudayalaya | Harish MM |
| 132 | Medanta the Medicity | Yatin Mehta |
| 133 | Tulip Hospital | Divya Saxena |
| 134 | Nayati Medicity | Vipul Mishra |
| 135 | P.D Hinduja National Hospital And MRC | Rishi Kumar |
| 136 | Pushpawati Singhania Research Institute Multispeciality Hospital, Sheikhsarai Phase-2 | Simnt Kumar Jha |
| 137 | Ruby Hall Clinic | Prashant Sakhavalkar |
| 138 | S.L.Raheja Hospital - A Fortis Associate | Dnyaneshwar Diwane |
| 139 | Sanjeevan Hospital | Subhal Dixit |
| 140 | Saveetha Medical College And Hospital | Kalaiselvan |
| 141 | SCB Medical College Hospital | Manoranjan Pattnaik |
| 142 | Shri Ram Murti Smarak Institute of Medical Sciences | Lalit Singh |
| 143 | Shubh Hospital | Fareed Khan |
| 144 | Sir H. N Reliance Foundation Hospital | Mehul Shah |
| 145 | Sugam Hospital | Prasanna |
| 146 | Tagore Hospital And Heart Care | Ziokov Joshi |
| 147 | Tata Memorial Hospital | Sheila Ninan Myatra / Manoj Gorade |
| 148 | Terna Speciality Hospital and Research Centre | Bharat G Jagiasi |
| 149 | Villoo Poonawalla Memorial Hospital | Amol Hartalkar |
| 150 | Yashoda Hospital, Malakpet Branch | B Saroj Kumar Prusty |
| 151 | Yashoda Hospital, Somajiguda Branch | Yogesh |

**Indonesia**

| **Site number** | **Hospital name** | **Investigator/Co-investigator** |
| --- | --- | --- |
| 152 | Memorial Hospital Murni Teguh | Ade Winata |
| 153 | ROI-IRD RSUD Dr. Soetomo | Maulydia |
| 154 | RS Infeksi Sulianti Saroso | Surya Oto Wijaya |
| 155 | RSUD Prof. Dr. Margono Soekarjo Purwokerto | Hermin Prihartini |
| 156 | RS Mayapada Jakarta | Shinta V. R. Hutajulu |
| 157 | RS Mayapada Tangerang | Rudy Manalu |
| 158 | RS Siloam Dhirga Surya | Ade Winata |
| 159 | RS. Adi Husada Undaan | Christrijogo Sumartono |
| 160 | RS. Advent | Reza Widyanto Sudjud |
| 161 | RS. Azra | Chrisma Adryana Albandjar |
| 162 | RS. Bhakti Dharmahusada | Ira Pitaloka |
| 163 | RS. St. Boromeus | Dewi Kusumawati |
| 164 | RS. Dr. Moewardi | Arifin |
| 165 | RSUP.Dr. Sardjito | Akhmad Yun Jufan |
| 166 | RSUD. Dr. Soetomo Surabaya | Bambang Pujo Semedi |
| 167 | RS. Eka Hospital BSD | Vanessy Theodora Silalahi |
| 168 | RS. Haji | Yudianto |
| 169 | RS. Hasan Sadikin | Erwin Pradian |
| 170 | RS. Hj. Adam Malik | Achsanuddin Hanafie |
| 171 | RS. Husada Utama | Mariza Fitriati |
| 172 | RS. Immanuel | Tinni Trihartini Maskoen |
| 173 | RS. Islam Makassar | Satriawan Abadi |
| 174 | RS. Jogjakarta Islam Hospital | Calcarina Fitriani Retno Wisudarti |
| 175 | RS. Karyadi | Johan Arifin |
| 176 | RS. Melinda 2 | Reza Widyanto Sudjud |
| 177 | RS. Mitra Keluarga Kenjeran | Prananda Surya Airlangga |
| 178 | RS. Pantiwilasa | Rupi'i |
| 179 | RSUP. Sanglah | I Made Wiryana |
| 180 | Santosa Hospital Bandung Central | Anang Achmadi |
| 181 | Siloam Hospital Jambi | Patra Rijalul Harly |
| 182 | Siloam Hospital Surabaya | Edward Kusuma |
| 183 | RS. Siloam-Karawaci | Primartanto Wibowo |
| 184 | RS. St. Elizabeth | Ade Veronica HY |
| 185 | RS. Telogorejo | Jeni Sarah Mandang |
| 186 | RS. Tugurejo | Meriwijanti |
| 187 | RS. Universitas Badung | I Wayan Aryabiantara |
| 188 | RS. Wahidin Sudirohusodo | Faisal Muchtar |
| 189 | RS. Zainoel Abidin Aceh | Fachrul Jamal Isa |
| 190 | RS. Cipto Mangunkusumo | Dita Aditianingsiih |
| 191 | RSK St Vincentius a Paulo | Nicolaas Parningotan Simamora |
| 192 | RSPAD Gatot Soebroto | Moch. Hasyim |
| 193 | RSU Kasih Ibu | I Gusti Putu Manuaba |
| 194 | RSUD Arifin Ahmad | Novita Anggraeni |
| 195 | RSUD Banyumas | Rudy Ariyanto Sanoesi |
| 196 | RSUD Brebes | Arief Munandar |
| 197 | RSUD Jayapura | Duma Saurma Siahaan |
| 198 | RSUD Pasar Minggu | Sri Rachmawati |
| 199 | RSUD Ulin | Oky Susianto |
| 200 | RSUP dr. M. Djamil | Liliriawati Ananta Kahar |
| 201 | RSUP Dr. Mohammad Hoesin | Zulkifli |
| 202 | RSUP Prof.Dr.R.D.Kandou | Mordekhai Leopold Laihad |

**Japan**

| **Site number** | **Hospital name** | **Investigator/Co-investigator** |
| --- | --- | --- |
| 203 | Chiba University Hospital | Nakada Takaaki |
| 204 | Fujita Health University Hospital | Yoshitaka Hara / Osamu Nishida |
| 205 | Hiroshima City Hospital | Kenji Uehara / Makoto Takatori |
| 206 | Hiroshima University Hospital | Shinichiro Ohshimo / Kazuya Kikutani / Nobuaki Shime |
| 207 | Jichi Medical University Hospital | Shin Nunomiya / Shinshu Katayama |
| 208 | Kagoshima University Medical and Dental Hospital | Bengo Atari/ Takashi Ito/ Yasuyuki Kakihana |
| 209 | Kameda Medical Center | Kohei Takimoto |
| 210 | Kobe City Medical Center General Hospital | Machi Yanai |
| 211 | Kobe University Hospital | Moritoki Egi |
| 212 | Kochi Medical School Hospital | Tomoaki Yatabe |
| 213 | Musashino RedCross Hospital | Yuki Kishiara |
| 214 | Nagasaki University Hospital | Ushio Higashijima / Motohiro Sekino |
| 215 | Nara Prefecture General Medical Center | Kazuaki Atagi |
| 216 | Osaka University Hospital | Hiroshi Ogura / Tsunehiro Matsubara |
| 217 | Shonan Kamakura General Hospital | Tadashi Kamio |
| 218 | St. Marianna University School of Medicine Hospital | Shigeki Fujitani |
| 219 | St. Marianna University School of Medicine, Yokohama City Seibu Hospital | Toru Yoshida |
| 220 | The Jikei University Hospital | Yukari Aoyagi / Shigehiko Uchino |
| 221 | Tohoku University Hospital | Masatsugu Hasegawa |
| 222 | Tokushima University Hospital | Jun Oto |
| 223 | Tokyobay UrayasuIchikawa Medical Center | Naoki Yamaguchi |
| 224 | University of Tsukuba Hospital | Yuki Enomoto |
| 225 | Yamagata University Hospital | Masaki Nakane |

**Kazakhstan**

| **Site number** | **Hospital name** | **Investigator/Co-investigator** |
| --- | --- | --- |
| 226 | Actobe, City Hospital | Amirova GS |
| 227 | Almaty Multispecial clinical hospital | Murat Daribaev |
| 228 | Central City Hospital. Stepnogorsk | Markov Viktor Evgenievich |
| 229 | City Clinical Hospital No. 1, Nur-Sultan | Vorobiev A A / Andrushenko A V |
| 230 | City Clinical Hospital No. 2, Nur Sultan | Aliya Torpakbaeva |
| 231 | Multispecial Medical Hospital No. 3, Nur-Sultan | Konkayeva M E |
| 232 | Kostanay, Oncological Hospital | Galkin A V |
| 233 | National Research Institute of Traumatology and Orthopedics | Ostanin P A |

**Laos**

| **Site number** | **Hospital name** | **Investigator/Co-investigator** |
| --- | --- | --- |
| 234 | Mahosot Hospital | Khamsay Detleuxay |

**Malaysia**

| **Site number** | **Hospital name** | **Investigator/Co-investigator** |
| --- | --- | --- |
| 235 | Hospital Kuala Krai, Kelantan | Noryani Mohd Samat |
| 236 | Hospital Kuala Lumpur, Malaysia | Ismail Tan |
| 237 | Hospital Melaka, Melaka | Nahla Irtiza Ismail |
| 238 | Hospital Pulau Pinang, Penang | Chew Har Lim |
| 239 | Hospital Raja Perempuan Zainab II, Kota Bharu, Kelantan | Wan Nasrudin Wan Ismail |
| 240 | Hospital Raja Permaisuri Bainon, Ipoh, Perak | Siti Rohayah Sulaiman |
| 241 | Hospital Sibu, Serawak | Anita Alias / Joanne Tiong Jia Wen |
| 242 | Hospital Sultan Ismail, Johor Bharu | Azmin Huda Abdul Rahim |
| 243 | Hospital Sultanah Bahiyah, Alor Setar, Kedah | Asmah Zainudin |
| 244 | Hospital Sultanah Nur Zahirah | Nik Azman Nik Adib |
| 245 | Hospital Tengku Ampuan Afzan, Kuantan | Zihni Abdullah |
| 246 | Hospital Tengku Ampuan Rahimah, Klang, Selangor | Hafizah |
| 247 | Hospital Universiti Sains Malaysia, Kelantan | Mohd Zulfakar Mazlan |
| 248 | International Islamic University Medical Centre, Kuantan, Pahang | Mohd Basri Mat Nor |

**Mongolia**

| **Site number** | **Hospital name** | **Investigator/Co-investigator** |
| --- | --- | --- |
| 249 | Bayangol Distric hospital of Mongolia | Munkhasiakhan |
| 250 | Bayanzurkh District Hospital of Mongolia | Naranpurev |
| 251 | Central Military Hospital of Mongolia | Naranpurev |
| 252 | Shastin Third Hospital of Mongolia | Munkhasiakhan |
| 253 | Songinokhairkhan District Hospital of Mongolia | Naranpurev |
| 254 | The First Central Hospital of Mongolia | Munkhasiakhan |
| 255 | The State Second hospital of Mongolia | Munkhasiakhan |

**Myanmar**

| **Site number** | **Hospital name** | **Investigator/Co-investigator** |
| --- | --- | --- |
| 256 | 300 bedded Teaching Hospital | Cho Myint Tun |
| 257 | Central Women Hospital | Thinzar Maw / Cho Cho |
| 258 | Insein General Hospital | Han Sein / Myo Malar Win |
| 259 | Ma-Gywe General Hospital | Lwin Lwin Hnin / Cho Cho Lwin |
| 260 | Mandalay General Hospital | Aye Su Mon / Yi Sandar Thein |
| 261 | Min-Bu General Hospital | Khin Le Le Yi |
| 262 | Nay Pyi-Taw, 1000 bedded Hospital | Myo Min Naing / Nu Nu May |
| 263 | New Yangon General Hospital | Lun Naing / Khin Saw Yu Aung |
| 264 | North Okkalapa General Hospital | Moe Thu Lin / Aung Kyi |
| 265 | Than- Lyin General Hospital | Kyaw Min Min Tun |
| 266 | Thin-Gan-Gyun Hospital | Suu New Khin / Khin Pyone Yi |
| 267 | Women Hospital Mandalay | Khin May Waan / Moe Thidar |
| 268 | Yangon General Hospital | Kyi Kyi Sann / Mu Mu Naing / Win Win Mar / Naing Naing Lin |

**Nepal**

| **Site number** | **Hospital name** | **Investigator/Co-investigator** |
| --- | --- | --- |
| 269 | Birat Medical College | Lalit Rajbanshi |
| 270 | Grande International Hospital | Trishant Limbu |
| 271 | Kathmandu Medical College Teaching Hospital | Baburaja Shrestha / Ujma Shrestha |
| 272 | Kathmandu University School of Medical Sciences | Ashish Shrestha |
| 273 | KIST Medical College | Rosi Pradhan |
| 274 | National Academy of Medical Sciences | Ravi Ram Shrestha |
| 275 | Nepal Medical College Teaching Hospital | Sulav Acharya |
| 276 | Om Hospital and Research Centre | Pramesh Sunder Shrestha |
| 277 | Shree Birendra Hospital | Puja Thapa Karki |

**Oman**

| **Site number** | **Hospital name** | **Investigator/Co-investigator** |
| --- | --- | --- |
| 278 | Armed Forces Hospital | Moosa Awladthani |
| 279 | Ibra Hospital | Jacob Paul |
| 280 | Ibri Hospital | Nadia Al Badi |
| 281 | Khoula Hospital | Adil Al Kharusi |
| 282 | Nizwa Hospital | Khalil Al Kharousi |
| 283 | Royal Hospital | Sandeep Kantor |
| 284 | Rustaq Hospital | Yohannan John / Said Al Mandhari |
| 285 | Sohar Hospital | Geetha Jacob |
| 286 | Sultan Qaboos Hospital Salalah | Amr Muhammad Esmat |
| 287 | Sur Hospital | BMJ Shetty / Ahmed Mostafa |

**Pakistan**

| **Site number** | **Hospital name** | **Investigator/Co-investigator** |
| --- | --- | --- |
| 288 | Aga Khan University, Karachi (MICU) | Naveed Haroon Rashid |
| 289 | Aga Khan University, Karachi (SICU) | Muhammad Sohaib |
| 290 | Aziz Fatimah Hospital, Faisalabad | Sonia Joseph |
| 291 | Civil Hospital, Karachi (SICU) | Safia Zafar |
| 292 | Doctor’s Hospital, Lahore (SICU) | Ahmed Farooq |
| 293 | Lady Reading Hospital, Peshawar (SICU) | Muhammad Sheharyar Ashraf |
| 294 | National Hospital and Medical Center, Lahore (SICU) | Tanveer Hussain |
| 295 | North West General Hospital, Peshawar (SICU) | Muhammad Hayat |
| 296 | Patel Hospital, Karachi | Ataur Rehman |
| 297 | Pakistan Institute of Medical Sciences (PIMS), Islamabad (MICU) | Syed Muneeb Ali |
| 298 | Shaukat Khanum Memorial Cancer Hospital, Lahore | Saad ur Rehman |
| 299 | Ziauddin University Hospital, Karachi (MICU) | Ashok Kumar |

**Philippines**

| **Site number** | **Hospital name** | **Investigator/Co-investigator** |
| --- | --- | --- |
| 300 | Asian Hospital Medical Center | Aaron Hernandez |
| 301 | Northern Mindanao Medical Centre | Crystal Aperocho |
| 302 | Quirino Memorial Medical Center | Raymundo Resurreccion |
| 303 | The Medical City Iloilo | Debbie Noblezada-Uy |
| 304 | The Medical City Ortigas | Jose Emmanuel Palo |
| 305 | UST Medical Center MICU 1 | Julie Visperas |
| 306 | UST Medical Center |  |

**Saudi Arabia**

| **Site number** | **Hospital name** | **Investigator/Co-investigator** |
| --- | --- | --- |
| 307 | Aseer Central Hospital- Abha | Amer Asiri /Ali Beshabshi |
| 308 | King Abdulaziz Medical City-Jeddah | Fahad Al-Hameed / Ohoud Al Orabi |
| 309 | King Abdulaziz Medical City- Riyadh | Yaseen Arabi / Eman Al Qasim |
| 310 | King Abdullah medical city- Makkah | Masood Iqbal / Tharwat Aisa |
| 311 | King Fahad Hospital of the University- Dammam | Mohammed Saeed Al Shahrani / Laila Asonto |
| 312 | King Fahad Hospital - Al Madinah | Ayman Kharaba |
| 313 | King fahad medical city- Riyadh | Abdullah al Mutairi |
| 314 | King Faisal Specialist Hospital & Research Center- Jeddah | Khaild Al Ghamdi/ Lama Hefni |
| 315 | Prince Mohammed Bin Abdulaziz hospital- Al Madinah | Ahmad Al Qurashi |
| 316 | Prince Sultan Military Medical City - Riyadh | Galeb Al Makhlafi |

**Singapore**

| **Site number** | **Hospital name** | **Investigator/Co-investigator** |
| --- | --- | --- |
| 317 | Changi General Hospital MICU | Roshni Sadashiv Gokhale |
| 318 | Changi General Hospital SICU | Noelle Lim |
| 319 | Khoo Teck Puat MICU | Manjit Pawar |
| 320 | Khoo Teck Puat SICU | Venkatesan Kumaresh / Naville Chia Chi Hock |
| 321 | Ng Teng Fong General Hospital ICU | Tan Chee Keat / Tan Rou An / Jared De Souza |
| 322 | National University Hospital MICU | Andrew Li / Yip Hwee Seng / Jason Phua |
| 323 | National University Hospital SICU | Addy Tan YH |
| 324 | Singapore General Hospital MICU | Melvin Tay Chee Kiang |
| 325 | Singapore General Hospital SICU | Ng Shin Yi |
| 326 | Seng Kang General Hospital ICU | Ho Vui Kian / Kiran Sharma |
| 327 | Tan Tock Seng Hospital MICU | Sennen Lew |
| 328 | Tan Tock Seng Hospital SICU | Lee Rui Min |

**South Korea**

| **Site number** | **Hospital name** | **Investigator/Co-investigator** |
| --- | --- | --- |
| 329 | Chonnam National University Hospital | Do Wan Kim |
| 330 | Chungbuk National University Hospital | Yoon Mi Shin |
| 331 | Chungnam National University Hospital | Song-I Lee |
| 332 | Daegu Catholic University Hospital | Kyung Chan Kim |
| 333 | Dongguk University Ilsan Hospital | Yun-Seong Kang |
| 334 | Ewha Womans University Mokdong Hospital | Soo Hwan Lee |
| 335 | Gyeongsang National University Changwon Hospital | Ho Cheol Kim |
| 336 | Hallym University Kangnam Sacred Heart Hospital | Yun Su Sim |
| 337 | Hallym University Sacred Heart Hospital | Sunghoon Park |
| 338 | Hanyang University Guri Hospital | Tai Sun Park |
| 339 | Inje University Busan Paik Hospital | Hongyeul Lee |
| 340 | Inje University Sanggye Paik Hospital | Youjin Chang |
| 341 | Jeonbuk National University Hospital | Heung Bum Lee |
| 342 | Korea University Ansan Hospital | Je Hyeong Kim |
| 343 | Korea University Guro Hospital | Young Seok Lee |
| 344 | Kyung Hee University Hospital | Won Gun Kwack |
| 345 | Myongji Hospital | In Byung Kim |
| 346 | Seoul National University Boramae Hospital | Tae Yun Park |
| 347 | Seoul National University Bundang Hospital | Young Jae Cho |
| 348 | Seoul National University Hospital | Sang-Min Lee |
| 349 | Sungkyunkwan University Samsung Medical Center | Kyeongman Jeon |
| 350 | The Catholic University of Korea, St. Mary's Hospital | Jongmin Lee |
| 351 | The Catholic University of Korea, St. Vincent’s Hospital | Shin Young Kim |
| 352 | Ulsan University Asan Hospital | Jin-Won Huh |
| 353 | Ulsan University Ulsan Hospital | Jong Joon Ahn |
| 354 | Yonsei University Gangnam Severance Hospital | Jae Hwa Cho |
| 355 | Yonsei University Wonju Severance Christian Hospital | Won-Yeon Lee |

**Taiwan**

| **Site number** | **Hospital name** | **Investigator/Co-investigator** |
| --- | --- | --- |
| 356 | MICU, Chiayi Chang Gung Memorial Hospital | Chin-Kuo Lin |
| 357 | MICU, CiShan Hospital | Chang-Ke Chu |
| 358 | MICU, E-D Cancer hospital | Jiun-Ting Wu |
| 359 | MICU 2, Kaohsiung Chang Gung Memorial Hospital | Chiung-Yu Lin |
| 360 | MICU 3, Kaohsiung Chang Gung Memorial Hospital | Yu-Mu Chen |
| 361 | MICU 5, Kaohsiung Chang Gung Memorial Hospital | Kuo-Tung Huang |
| 362 | MICU,  LinKo Chang Gung Memorial Hospital | Han-Chung Hu |
| 363 | MICU, National Cheng Kung University Hospital | Cong-tat Cia |
| 364 | MICU, National Taiwan University Hospital | Jung-Yien Chien |
| 365 | MICU, Taichung Veterans General Hospital | Chun-Te Huang |
| 366 | RICU, Taichung Veterans General Hospital | Pin-Kuei Fu |

**Thailand**

| **Site number** | **Hospital name** | **Investigator/Co-investigator** |
| --- | --- | --- |
| 367 | King Chulalongkorn Memorial Hospital MICU | Nattachai Srisawas |
| 368 | King Chulalongkorn Memorial Hospital SICU | Manasnun Kongwibulwut |
| 369 | Maharaj Nakorn Chiang Mai Hospital SICU | Kaweesak Chittawatanarat |
| 370 | Srinagarind Hospital MICU-I | Worapot Daewtrakulchai / Anakapong Phunmanee |
| 371 | Srinagarind Hospital MICU-II | Anupol Panitchote / Boonsong Patjanasoontorn |
| 372 | Vajira Hospital | Chaiwut Sawawiboon |

**Vietnam**

| **Site number** | **Hospital name** | **Investigator/Co-investigator** |
| --- | --- | --- |
| 373 | 115 People Hospital | Lê Minh Trung |
| 374 | Bach Mai Hospital | Đỗ Ngọc Sơn |
| 375 | Bai Chay Hospital | BS Bùi Nhật Hà |
| 376 | Can Tho Central Hospital | Dương Thiện Phước |
| 377 | Cho Ray Hospital | Huỳnh Quang Đại |
| 378 | Da Nang Hospital | Nguyễn Tấn Hùng |
| 379 | Dong Da Hospital | Lê Thị Phương Thúy |
| 380 | Ha Noi Medical University Hospital | Hoàng Bùi Hải |
| 381 | Hue Central Hospital | Hoàng Trọng Ái Quốc |
| 382 | Saint Paul Hospital | Trần Hoài Linh |
| 383 | Thai Nguyen Central Hospital | Vũ Hải Yến |
| 384 | Thanh Nhan Hospital | Phạm Trà Giang |
| 385 | Viet Tiep Hospital | Nguyễn Thị Ngà |
| 386 | VINMEC International Hospital | Nguyễn Đăng Tuân |
